# ARH-seq: identification of differential splicing in RNA-seq data

**DOI:** 10.1093/nar/gku495

**Published:** 2014-06-11

**Authors:** Axel Rasche, Matthias Lienhard, Marie-Laure Yaspo, Hans Lehrach, Ralf Herwig

**Affiliations:** Max-Planck-Institute for Molecular Genetics, Department of Vertebrate Genomics, Ihnestrasse 63-73, 14195 Berlin, Germany

## Abstract

The computational prediction of alternative splicing from high-throughput sequencing data is inherently difficult and necessitates robust statistical measures because the differential splicing signal is overlaid by influencing factors such as gene expression differences and simultaneous expression of multiple isoforms amongst others. In this work we describe ARH-seq, a discovery tool for differential splicing in case–control studies that is based on the information-theoretic concept of entropy. ARH-seq works on high-throughput sequencing data and is an extension of the ARH method that was originally developed for exon microarrays. We show that the method has inherent features, such as independence of transcript exon number and independence of differential expression, what makes it particularly suited for detecting alternative splicing events from sequencing data. In order to test and validate our workflow we challenged it with publicly available sequencing data derived from human tissues and conducted a comparison with eight alternative computational methods. In order to judge the performance of the different methods we constructed a benchmark data set of true positive splicing events across different tissues agglomerated from public databases and show that ARH-seq is an accurate, computationally fast and high-performing method for detecting differential splicing events.

## INTRODUCTION

Alternative splicing is an effective cellular mechanism that allows to generate multiple protein isoforms from a single nuclear ribonucleic acid (RNA) template, and the expression of specific splice forms is crucial for development, differentiation and disease processes (1). Dissecting the biological importance of alternative splicing is still a difficult task since it has been shown that much of the messenger RNA diversity observed includes low-abundance transcripts that are consequences of stochastic noise in the splicing machinery and have no functional significance (2,3). Even for robust isoform differences detailed experimental analysis of individual isoforms is required what makes prediction of alternative splicing and its validation rather complicated (4).

Predictions of the proportion of human genes expressed with more than one isoform increased with the granularity of technology development in recent years. While previous functional studies estimated the amount of genes that undergo alternative splicing at a rate of 40–60% the advent of new high-throughput experiments, in particular RNA-seq, has increased this rate to over 90% (5,6).

Several methods for computational prediction of alternative splicing have been proposed that are based on the quantification of RNA-seq data (7). Methods can be classified into those that use existing genome annotation in order to quantify known isoforms (8) and those that allow *de novo* detection of isoforms from the genomic mapping of the short reads (9,10). Furthermore, methods are either based on isoform- or exon-wise analysis. DASI has been one of the first methods proposed specifically for RNA-seq analysis. Similar to DEXSeq it is based on expression evaluation at the exon-level and uses a statistical test to assess splicing (11,12). cuffdiff allows expanding from known annotation by generating transcripts from tophat alignments and subsequently comparing these transcripts between conditions (9,10). MISO and MATS use Bayesian approaches taking into account only the reads discriminating between different isoforms (13,14). cuffdiff, MISO and MATS incorporate the concept of junction reads in their computations, i.e. reads that bridge neighbouring exons. Splicing Index was originally developed for exon microarrays and is an expression-based measure that has been adapted to RNA-seq analysis (15). Similarly, PAC was proposed for analysing exon microarrays with a different correction for gene expression (16). Correlation of expression between conditions was proposed for splicing predictions using a heuristic sampling measure (17).

In this work we describe ARH-seq, a new method for predicting differential splicing of genes in case–control studies that is based on the information-theoretic concept of entropy. We build on the existing method ARH that was previously tested and validated in the context of exon microarrays (18). ARH has already been proven as a robust and reliable method for detecting differential splicing biomarkers for example in the context of cancer progression (19). The method bases on known exon annotation and predicts differential splicing of the corresponding gene by transforming the exon expression changes into a probability distribution that is subsequently evaluated with statistical entropy. We extended the method to high-throughput sequencing data by combining exon and exon junction expression quantification derived from digital read counts and by adaptation of the subsequent statistical analysis.

We show that ARH-seq has several inherent features, for example independence of differential gene expression as well as independence of the number of exons per gene, that make it particularly suited for differential splicing analyses. We applied the method to publicly available RNA-seq data derived from human tissues (Illumina Human Body Map 2.0) (5,6,20) as well as additional Affymetrix exon microarrays (20) and polymerase chain reaction (PCR)-validated data sets (21). By summarizing differential splicing scores computed from pairs of human tissues, we show that ARH-seq can be parameterized robustly so that the significance of differential splicing is quantified.

In order to test and challenge our workflow we conducted a comprehensive comparison of ARH-seq with eight other published methods described above. To address the lack of validation data and to be able to compare the performance of the different methods we agglomerated benchmark data from public databases that contains known differential splicing of genes with respect to 13 different human tissues (22). By ordering the computational predictions derived from the comparison of the tissue expression data sets in decreasing splicing indication these true positive sets constituted classiﬁers that allowed visualizing the performance of the individual methods with the receiver operating characteristic (ROC). The ROC performance was quantiﬁed with the area under the curve (AUC). The comparison shows that ARH-seq is a highly competitive and accurate computational method for predicting differential splicing with efficient runtime performance and high throughput.

## MATERIALS AND METHODS

### Benchmark sequencing data

The value of benchmark data sets for methods development and comparison is known (23,24). Benchmark data refers to solid and accurate experimental data as well as sets of true positive data that can be used to judge performance of the computational methods. As experimental data we chose three publicly accessible data sets: The ‘Illumina 50f’ data were taken from a 50-bp paired-end data set generated from different human tissues (E-MTAB-513 or GSE30611). Since we did not use the paired-end information in this workflow we only considered forward reads in the analysis and denoted this with an ‘f’. The ‘Illumina 75’ data refer to 75 bp reads generated from the same tissues and follow single-read transcriptome protocols recommended by the supplier. The ‘Illumina 32’ data were the union of two published data sets comprising a subset of seven tissues (5,6).

### Short read alignment

Sequencing reads were aligned using a two-step procedure. In the first step, reads were aligned according to University of California Santa Cruz browser (UCSC) (25) HG19 genome annotation with bowtie allowing multiple alignments per read. Only reads located completely within Ensembl 58 exons were used for quantification. In the second step, reads that could not be aligned to the genome sequence were re-aligned to exon junctions. Here again multiple mapping of reads was allowed. The ARH-seq workflow utilized ‘synthetic’ junctions generated for all non-overlapping exon pairs in a gene. The ‘synthetic’ junction is composed of two concatenated sequences of three-fourths times the read length from the flanking exons. With this rather conservative selection it was assured that a junction read aligns to an exon with at least one-fourth of its read length.

Bowtie was used because it generates fast alignments and because it is also the basis for the different junction aligners that were compared subsequently (version 0.12.7, 64-bit; parameters –strata -y –best –chunkmbs 128 -k 25 -m 25 -p 20 –sam) (26). To assess the influence of different junction alignment methods on the performance of differential splicing prediction we compared four methods: ‘synthetic’ junctions as described above, tophat (version 1.3.1), MapSplice (version 1.15) and SpliceMap (version 3352 linux-64) (9,27,28). For tophat the reads spanning the junctions were assigned to a junction identifier assembled from the corresponding Ensembl 58 exons (9). Then, junctions were filtered for exon identifiers matching the same gene.

### Exon and exon junction annotation

Gene structures were taken from BioMart using Ensembl 58 annotation for ‘exon to gene’ and ‘junction to neighbouring exons to gene’ mapping (29–31). A total of 49 733 genes and 533 087 exons could be retrieved. Since, for a particular gene, the number of possible exon junctions increases exponentially, we filtered for junctions where the second exon started at a higher genomic coordinate than the end of the first exon. Additionally, we excluded single-exon genes. These filters resulted in 3 682 059 junctions in 32 414 genes.

### Exon and exon junction expression quantification

For each exon and for each junction window the number of aligned reads was counted separately per sample (case and control samples). Next reads per kilobase per million (RPKM) values were computed in order to provide normalized expression values (32). Exon RPKM values were computed in each sample by scaling the exon length to 1000 (exon end position minus exon start position) and scaling the read number to 1 000 000 (total number of aligned exon and junction read counts).

In order to make junction expression comparable to exon expression the exon junction RPKM values were computed like above using the size of the junction window instead of the exon length. For the additional junction alignment methods we used the following window lengths: (i) Tophat: 2*<read length>-10 assuming a seed of 5 bp in each exon; (ii) MapSplice: 2*<read length>-2; (iii) SpliceMap: 2*<read length>-20 (27,28). Tophat, MapSplice and SpliceMap were run with standard parameters, except for tophat on Illumina 32 where segment length was decreased and Ensembl annotation was passed (parameters -a 5 –GTF ensembl_annotation -p 20 –segment-length 16 -m 1).

### ARH-seq workflow

The ARH-seq method was applied as previously described (18). A schema of the workflow along with an example calculation is given in Supplementary Figure S1A. The mathematical formulation of the method is given in Supplementary Figure S1B. In order to translate abstract ARH-seq scores to *P*-values, a background distribution was fitted from the RNA-seq data (Supplementary Figure S2). ARH-seq values of all case–control studies from the benchmark data were summarized to compute a background distribution what resulted in a fit with the Weibull distribution with scale parameter of 0.18 (standard deviation of 0.00025) and shape 0.44 (0.00020) (33). It should be noted that the background distribution is rather selective since the *P*-values provided by ARH-seq were computed from case-control studies with very strong splicing indication (i.e. between different tissues). Thus, these *P*-values should be interpreted as rather conservative, and since splicing is a widespread mechanism in biology even larger *P*-values could still reflect a biologically valid splicing event.

### Methods comparison

We compared ARH-seq with eight alternative approaches: Splicing Index, PAC, Correlation, DASI, DEXSeq, cuffdiff, MISO and MATS. Exon-expression based splicing predictions have been developed for microarrays so far and we adapted three of these methods to RNA-seq data: Splicing Index, PAC and Correlation (16,17,34). Partly, these methods have already been applied in the context of sequencing data. The methods were individually tested with raw count, RPKM and combined exon-junction expression and the best performing quantification was used in each case. Splicing Index and PAC are heuristic measures that compare the expression of individual exons with the average gene expression (16,34). Correlation uses the Pearson coefficient to assess the linearity of exon expression within the gene (17). DASI (Solas package version 0.2) and DEXSeq (version 1.4.0) apply Fisher and Chi^2^ tests for exon expression and deviation models incorporating different aspects like exon length or biological noise (11,12). cuffdiff (version 1.0.3) uses alignment-based isoform predictions and relates their abundances (10). MISO (version 0.4.9) computes a Bayesian Psi-factor for reads contrasting inclusion or exclusion of an exon for each sample and derives a Bayes-factor for differential analysis of samples (13). MATS (version 3.0.7) calculates a uniform prior over all exon-skipping events and feeds an MCMC model (14). MISO and MATS run with standard settings on Python 2.7.3.

### Validation data

To assess the performance of the methods it was necessary to establish a validation environment providing method independent true positive splicing events. For that we chose the manually curated database AEdb (35,36). An overview of the number of the identified splicing events is given in Supplementary Table S1. The AEdb sequence ﬂat ﬁle was downloaded (http://www.ebi.ac.uk/asd/aedb/) and the splicing events were ﬁltered by splicing mechanism (cassette exon events), species (human, mouse, rat) as well as the availability of a genetic sequence for the events. Thirteen different tissues were covered by AEdb with at least 10 true positive splicing events. We denoted a spliced exon as true positive event between two tissues when there was evidence for respective differential splicing in the database. Events attributed to these 13 tissues were selected and the corresponding sequences of the differentially spliced exons were aligned to human exon sequences from Ensembl 58 for exact matches. In a number of cases the alternative skipping or inclusion event was observed in more than one tissue. For tissue-speciﬁc comparisons, i.e. comparing a single tissue against all others, such events were skipped. For pairwise-tissue comparisons, i.e. comparing a tissue against another tissue, only the events of differential exon inclusion between the two tissues were used as true positive and it was additionally filtered for events where the exon is expressed in at least one of the two conditions.

### Post-processing

Candidates for differential splicing were selected based on post-processing steps ensuring (i) the sufficient read coverage in at least one condition (case or control) and (ii) significance of splicing indication. As reasonable setting taking into account the validation data we required exon/junction/gene expression with an RPKM value ≥0.75 in at least one condition and an ARH-seq *P*-value <0.2.

## RESULTS

### Characteristics of validation data set

While there are several tools available that test and validate methods for predicting differential splicing *in silico* (e.g. BEERS (37) and Mason (38)) experimentally validated benchmark data is rather rare. Thus, an important step in the workflow addressed the agglomeration of experimentally validated differential splicing events across different human tissues from public resources.

We extracted confirmed splicing events from the AEdb database (22) and retrieved 330 unique events associated with the 13 tissues under analysis (heart, liver, testis, kidney, prostate, thyroid, brain, skeletal muscle, lung, colon, blood, muscle and spleen). Lengths of the corresponding exons are depicted in Supplementary Figure S3. An important observation is that a fraction of exons is shorter than the actual read lengths so that no expression quantification could be calculated for these exons since we required full inclusion of the sequence read. Depending on the read lengths and tissues under analysis distinct subsets were selected as true positive sets for evaluation. The numbers of true positive splicing events for the various tissue comparisons are listed in Supplementary Table S1, and a summary statistic of the tissue data sets and validation events is shown in Table 1.

**Table 1.** Tissue data sets and true positive splicing events

Tissue	Mapping level	Illumina 32	Illumina 75	Illumina 50f	Affymetrix exon array	AEdb TP
Heart	Exon	26 497 133	90 096 333	152 095 841	x	13
	Junction	1 792 700	16 524 040	15 970 634		
Liver	Exon	33 764 463	111 467 996	147 140 423	x	40
	Junction	2 974 503	30 096 758	16 596 701		
Testis	Exon	49 450 100	112 055 446	117 648 767	x	62
	Junction	4 610 852	27 751 282	13 021 071		
Breast	Exon	na	98 295 674	160 271 222	x	x
	Junction	na	19 416 413	18 377 157		
Kidney	Exon	na	102 936 196	159 845 509	x	35
	Junction	na	18 726 028	1 7260 878		
Prostate	Exon	na	137 440 809	103 076 631	x	11
	Junction	na	27 591 808	11 317 574		
Thyroid	Exon	na	106 721 876	143 112 126	x	10
	Junction	na	26 471 132	25 146 417		
Brain	Exon	25 394 892	75 843 985	145 012 531	na	148
	Junction	1 792 406	14 036 937	16 148 859		
Skeletal muscle	Exon	35 937 904	111 017 485	113 197 592	na	15
	Junction	2 857 497	26 281 433	12 503 218		
Lung	Exon	29 783 930	128 222 626	123 907 913	na	22
	Junction	2 330 613	26 039 119	13 785 738		
Colon	Exon	50 295 406	117 311 390	129 051 270	na	13
	Junction	3 467 812	20 682 248	17 143 662		
Adipose	Exon	na	103 017 625	131 804 299	na	x
	Junction	na	21 068 190	18 053 743		
Adrenal	Exon	na	97 252 603	133 042 562	na	x
	Junction	na	19 144 480	18 230 504		
Lymph node	Exon	na	124 624 185	115 655 278	na	x
	Junction	na	24 067 126	12 011 253		
Ovary	Exon	na	118 850 487	121 800 678	na	x
	Junction	na	24 972 481	12 456 813		
Blood	Exon	na	135 567 404	144 373 657	na	18
	Junction	na	29 120 343	13 847 002		
Cerebellum	Exon	na	na	na	x	x
	Junction	na	na	na		
Muscle	Exon	na	na	na	x	36
	Junction	na	na	na		
Pancreas	Exon	na	na	na	x	x
	Junction	na	na	na		
Spleen	Exon	na	na	na	x	20
	Junction	na	na	na		
No. of pairwise tissue comparisons		21	45	45	28	x
No. of tissue specific comparisons		4	7	7	8	

Tissue data sets used in performance assessment. ‘Illumina 32’ is the union of two published data sets utilizing 32bp sequencing reads. ‘llumina 75’ and ‘Illumina 50f*’* correspond to the HiSeq BodyMap V2.0 data set with 75 and 50 bp (forward) reads. Affymetrix exon array tissue data is available from the Affymetrix homepage. In the rightmost column the number of true positive splicing events from AEdb is shown. The last two rows give the number of pairwise-tissue and tissue-specific test cases which could be generated for the data sets. Abbreviations: TP, true positive splicing events; pw, pairwise; ts, tissue specific; jctn, junction; na, x, no data available.

It is observable that the AEdb database shows a tendency towards genes with many exons compared to the overall exon number per gene (Supplementary Figure S4). This is explainable since the annotated splicing events were derived from targeted functional studies where multi-exon genes were investigated rather than genes with low exon number.

### Exon and exon junction quantification

RPKM normalization was used to correct the exon read counts for exon length and experiment-wise sequencing depth (32). Next, we examined differences in the quantification of exon junctions with tophat, MapSplice and SpliceMap alignments (9,27,28) and found that these alignments showed only little variation with respect to their impact on splicing prediction (see Supplementary Figure S5). To take full advantage of the RNA-seq data we combined exon with exon junction quantification exploring a total number of 3 682 058 junctions in 32 414 genes. By concatenating the exon sequences adjacent to a junction, a ‘synthetic’ junction sequence was generated. For the ARH-seq approach the exon sequence snippet was sized to 75% of the read length resulting in a ‘synthetic’ junction sequence of 1.5 times the read size. Only reads not aligning to exons were re-aligned to the junction sequences. Supplementary Figure S6 shows the effect on splicing prediction with different junction window sizes.

**Figure 1.**
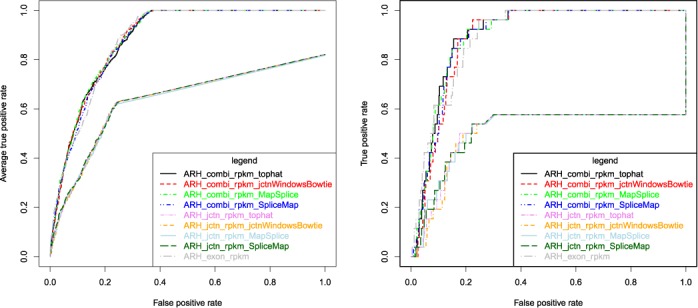
Impact of alignment and read counting. ROC curves for differential splicing prediction comparing different junction alignment variants with respect to AEdb conﬁrmed splicing events. Junction expression was computed with tophat (marked ‘_tophat’), MapSplice (marked ‘_MapSplice’), SpliceMap (marked ‘_SpliceMap’) and ‘synthetic’ junction windows (marked ‘_jctnWindowsBowtie’). Identified splice sites were mapped to Ensembl-annotated genes. ARH-seq predictions based solely on junction expression (marked ‘_jctn_’), exon expression (marked ‘_exon_’) and combination of both (combi-counts, marked ‘_combi_’) were compared. The left plot shows averaged pairwise tissue evaluations and the right plot the evaluation of the brain versus liver scenario with the ‘Illumina 75’ data set.

Since the number of combinatorial possible junctions in the genome exceeds by far the number of known expressed transcripts it is expectable that only a fraction of junctions is experimentally measurable. But even for the 156 annotated differential exon inclusion events between brain and liver tissues the neighbouring junctions of 105 exons had no matching sequence read. This result gives evidence that by far the most informative part for differential splicing prediction is contributed through exon quantification, though a slight increase in performance is observable when combining exon with exon junction quantification. On the other hand, splicing prediction solely based on junction information showed poor performance (Figure 1).

### ARH-seq characteristics

Based on the observations above we have used a combination of exon and exon-junction quantification—the combi-counting. The basic idea here was to scale up quantification of each exon with an additional factor that was determined by the quantification of its respective possible junctions. These combi-counts were defined as the sum of the exon RPKM values and the neighbouring junction RPKM values at both ends. For example, for a gene with four exons, and corresponding RPKM values *e_1_*, *e_2_*, *e_3_*, *e_4_*, the second exon *e_2_* has possible junction RPKM values *j_12_*, *j_23_* and *j_24_* and the combi-count is defined as *c_2_ = e_2_ + j_12_ + j_23_ + j_24_*. It should be noted that the combi-counts up-weight expression changes caused by a splicing event. When an expressed transcript *t_1_* = (*e_1_*, *e_2_*, *e_3_*, *e_4_*) in sample *x* changes to a transcript *t_2_* = (*e_1_*, *e_3_*, *e_4_*) in sample *y*, with one exon skipping event in exon *e_2_*, this event changes expression of *e_2_* and of all neighbouring junctions *j_12_*, *j_23_* and *j_24_*. Using combi-count *c_2_*, the expression changes are amplified compared to the normal biological and technical expression variation and the prediction is more stable even if some junctions have no matching reads, for example because of insufficient sequencing depth or/and poor library complexity. For the computation of combi-counts, it is necessary to have comparable expression values what necessitates the use of RPKM (or similar values) for exon and exon junction quantification.

**Figure 2.**
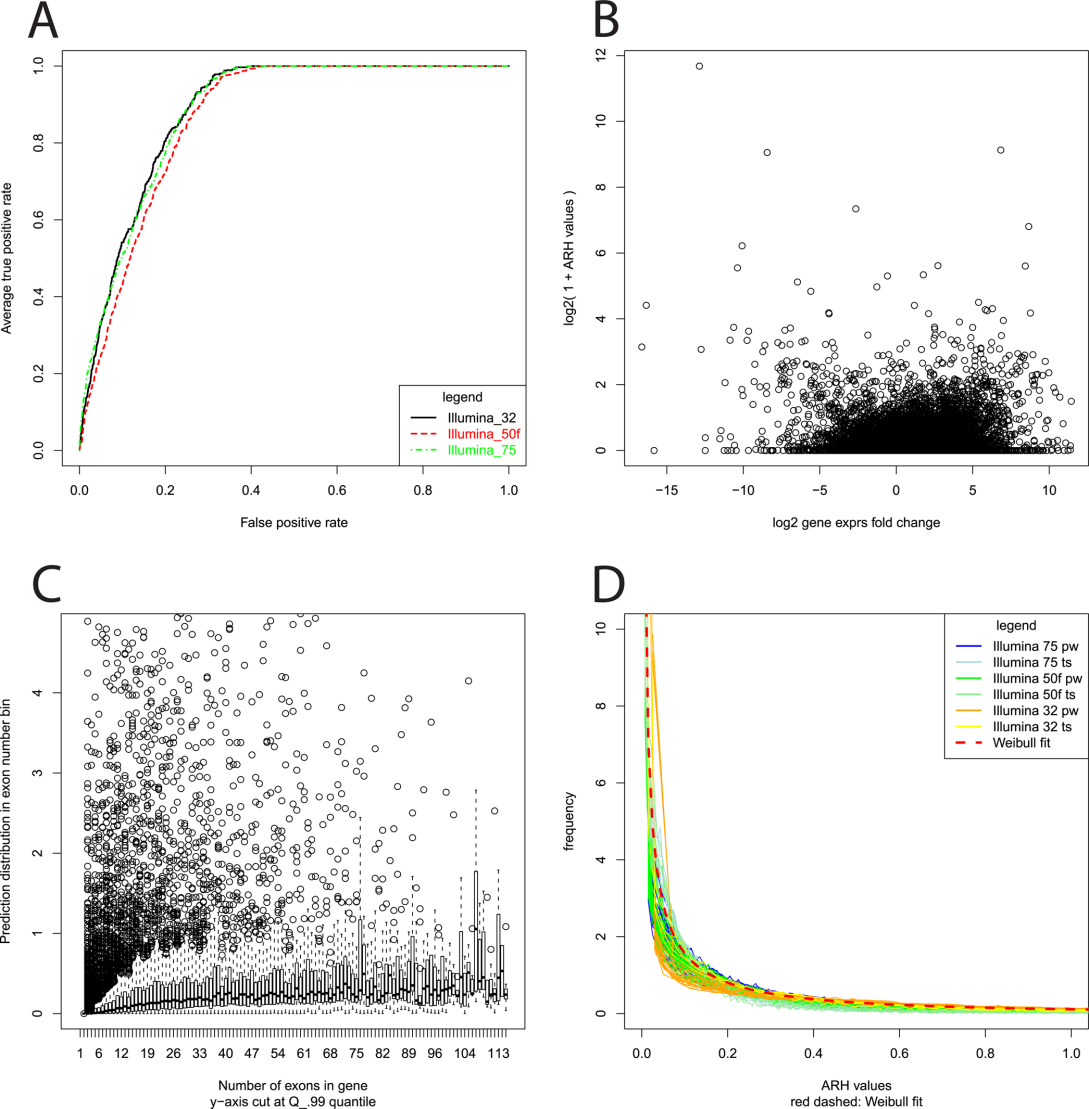
ARH-seq characteristics. (**A**) ARH-seq prediction performance for pairwise tissue comparisons on data sets generated with different sequence read lengths. (**B**) ARH-seq predictions (*y*-axis) versus gene expression changes (log_2_-scale; *x*-axis) in brain versus liver comparison. (**C**) ARH-seq predictions (*y*-axis) versus gene exon number (*x*-axis). All genes with the same exon number were summarized and according box plots of ARH-seq values for brain versus liver are shown. (**D**) Distribution of ARH-seq values plotted for all sequencing data sets. The resulting Weibull fit is superimposed as dashed line.

ARH-seq evaluates the distribution of changes in exon combi-counts by comparing two samples with the information-theoretic concept of entropy (an illustrative example is given in Supplementary Figure S1A; the formalization is given in Supplementary Figure S1B). The output of ARH-seq for a pair of conditions is a *P*-value at the gene level indicating whether the gene is differentially spliced or not. Additionally, for each exon ARH-seq computes a numerical value that indicates its differential splicing probability so that exons within the gene can be prioritized. The method has several inherent features that make it useful for such predictions as visualized in Figure 2. Firstly, ARH-seq is independent of the length of sequence reads (Figure 2A), expression fold-changes (Figure 2B) and gene exon numbers (Figure 2C) which are factors that commonly bias alternative splicing prediction methods. In particular the ability of predicting differential splicing in scenarios where differential expression is observed is an advantage. Secondly, ARH-seq does not make any *a* *priori* assumption that restricts the number of transcripts to process, e.g. a uniform distribution of sequence read counts along the exons of a gene, or a specific expression probability background model. Additionally, ARH-seq values can be parameterized. We used the experimental data to fit the distribution of ARH-seq to a Weibull distribution in order to judge significance of the observations and could show that this fit is fairly robust across the different experimental data sets (Figure 2D).

### Performance of computational methods

An overview of methods for analyzing RNA-seq data is summarized in (7). For our comparison, three methods were adapted from exon arrays to RNA-seq: Splicing Index, PAC and Correlation (16,17,34). Splicing predictions of the nine methods classify either in gene-based (ARH-seq, correlation, DASI, cuffdiff) or exon-based (Splicing Index, PAC, DEXSeq, MATS, MISO) predictions. Evaluations were computed on gene/exon level corresponding to the method with the true positive events mapped to respective identifiers. Extended analyses for all methods are shown with respect to their dependency on differential gene expression (Supplementary Figure S7) and on the number of exons per gene (Supplementary Figure S8).

**Figure 3.**
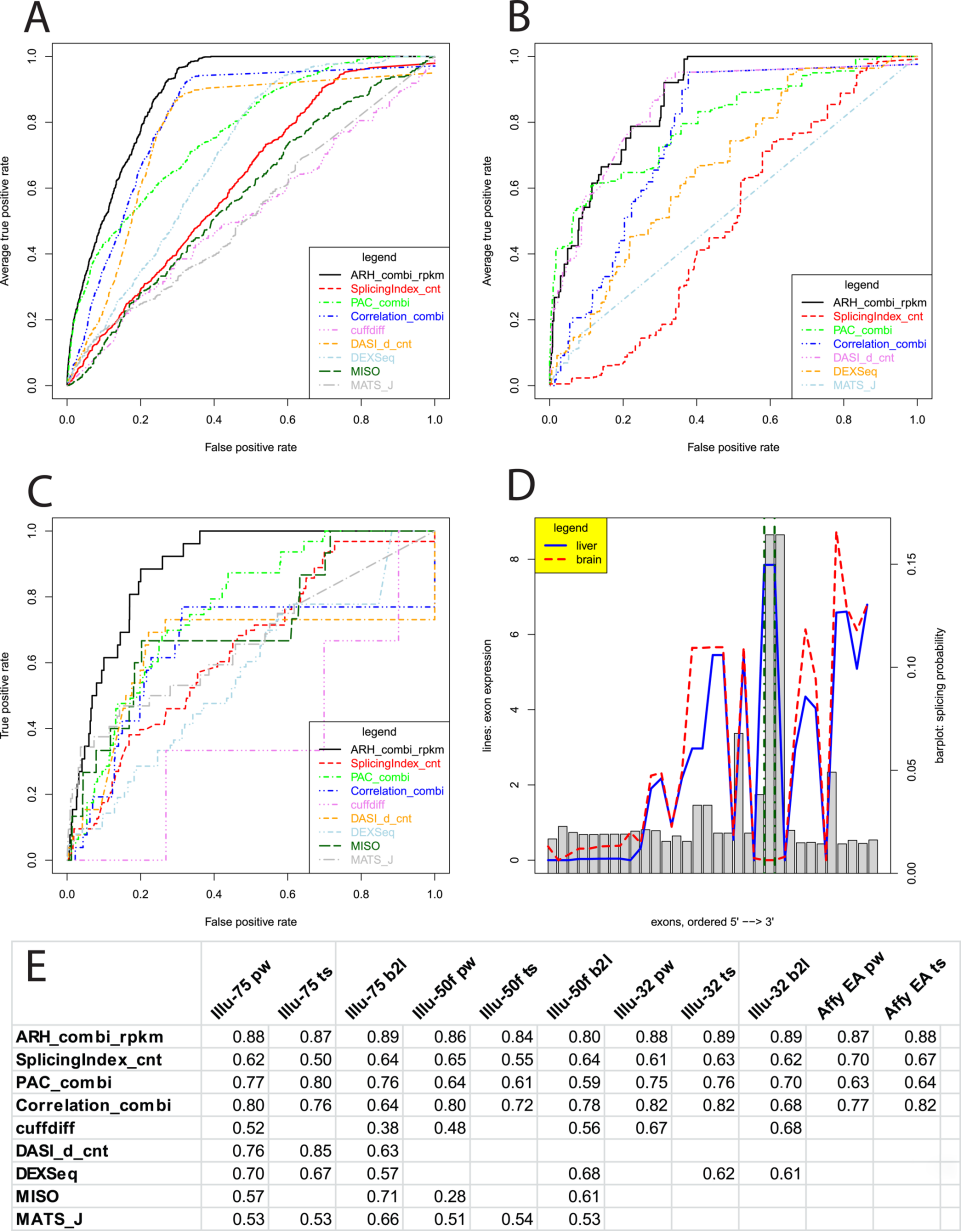
Methods comparison. (**A**) ROC curves for differential splicing prediction methods using ‘Illumina 75’ data set with all possible pairwise test cases (i.e. comparing one tissue against another tissue). (**B**) ROC curves assessing tissue-specific splicing events (i.e. comparing one tissue against all others). Due to highly variable sample sizes two methods had to be skipped. (**C**) ROC curves assessing differential splicing in brain versus liver. (**D**) Example of a detected true positive splicing event in the gene *MPZL1*. Exons are shown on the *x*-axis. RPKM values are visualized with the red dashed line for brain and blue solid line for liver. The splicing probabilities used for the entropy-based prediction are denoted as grey bars. Two exons known for splicing are marked with green dot-dashed lines. (**E**) AUC values for the different test cases including exon array results (pw = pairwise; ts = tissue specific; b2l = brain versus liver; EA = exon array data).

To challenge the methods we constructed several benchmarks with the RNA-seq and the validation data: (i) paired tissue comparisons (i.e. one tissue compared to another tissue) with a total number of 330 true positive cases (Figure 3A), (ii) tissue-specific comparisons (i.e. a single tissue compared to all others) (Figure 3B) and (iii) a selected paired tissue comparison using brain and liver (Figure 3C). The brain versus liver test case was chosen because the two tissues are known for many tissue specific splicing events. For this test case, we retrieved 156 exon skipping events in 67 genes from AEdb. From the ‘Illumina 75’ and ‘Illumina 50f’ data sets we extracted ten tissues with sufficient number of true positives for the paired tissue comparisons allowing for a total of 45 paired comparisons and seven tissue specific comparisons; from the ‘Illumina 32’ data set we extracted seven tissues with sufficient number of true positives allowing for 21 paired comparisons and four tissue specific comparisons; from the Affymetrix exon array data set we extracted eight tissues allowing 28 paired and eight tissue specific comparisons. For each method the predictions were sorted by decreasing splicing indication. Then, the positions of the true positives were identiﬁed in the ranked result lists. ROC was used for visualization using the ROCR-package version 1.0-4 in R (39). We observed ARH-seq as best performing method in all cases (Figure 3E). Complete results for all test cases are displayed in Supplementary Figure S9. The AUC of the ROC were computed with ROCR for quantiﬁcation of the performance and listed in Supplementary Table S2. Additionally, we calculated commonality tables in order to quantify the overlap of the different methods. In particular, assessing commonality among the top predictions of each method is a practical issue, for example for further validation studies. Commonality tables for the top 250 predictions are shown in Supplementary Tables S3 and S4. In spite of its intuitive simplicity and great acceptance it is worth mentioning that the ROC-curve based performance evaluation is affected by some method-dependent artefacts which are discussed in Supplementary Figure S10.

Furthermore, in order to exemplify the ability of ARH-seq to enable detailed identification of splicing events we recovered an exon skipping event in the gene *MPZL1* in brain compared to liver (*P* = 0.09) which was reported previously in the literature (Figure 3D and Supplementary Figure S11) (40).

Finally, PCR-based validation data were available for eleven different exons in muscle tissue (21). Results confirmed ARH-seq as best performing method (Supplementary Figure S12).

### Runtime performance

With the growing size of RNA-seq data, time efficiency of analysis workflows is an issue. The presented workflow allows to process two sequencing lanes (∼80 million 75 bp reads) within 3 h on a 32 core AMD Opteron 8360 SE computer with 128GB RAM running Linux-64. In detail, the different analysis steps needed the following runtimes: indexing of the genome reference for bowtie took 2 h and establishing the Ensembl mapping as local lookup table took 2.5 h. The alignment and count file calculations took <2 h for ‘Illumina 75’ and a few minutes for ‘Illumina 32’ per lane. Runtime for the junction alignments was 15 min for the synthetic junctions, 7 h for tophat, 13 h for SpliceMap and 25 h for MapSplice. Computation time for RPKM normalization, ARH-seq predictions as well as post-processing steps took a total of 45 min. ARH-seq computational predictions were computed within 2 min. Faster are Splicing Index, PAC and correlation that did computing within seconds. DASI needed 12–18 h, similar to DEXSeq with about 12 h. Both methods perform statistical tests for all exons/genes in R. cuffdiff evaluates two BAM files in 1 h (15 cores in parallel). MISO starts with bowtie BAM files preparing two samples in 100 min and performs differential analysis in 15 min however this extends to 12 h for paired-end analysis (six cores). MATS requires performing alignment with tophat in about 10 h per sample (eight cores) and the differential analysis in 2.5 h. It is, however, difficult to compare these runtimes because of different implementations. In summary, most of the runtime was attributed to the read alignment and the computation of the different algorithms varied with several orders of magnitude where time was generally not correlated with better prediction results.

## DISCUSSION

### ARH-seq runtime performance

**Figure 4.**
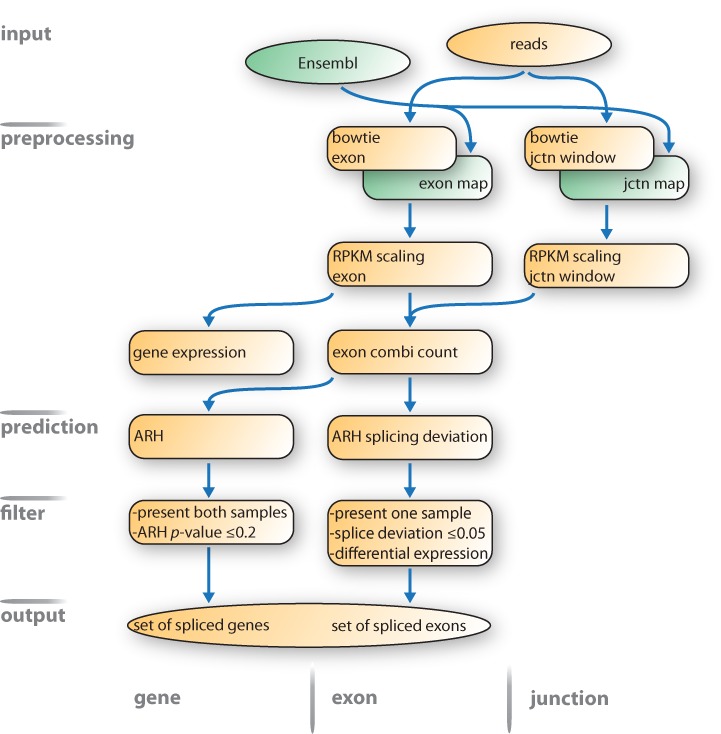
ARH-seq differential splicing prediction workflow. The proposed workflow starts with a set of sequencing reads and an Ensembl genome annotation and finally generates a set of spliced genes ordered by ARH-seq prediction scores. Reads are aligned to the genome with bowtie and counts are generated for exons and junction windows. Using RPKM-scaled values gene expression and combi-counts are calculated. Splicing prediction is performed with ARH-seq on the combi-counts. Spliced exons are judged by their splicing deviation. Finally, results are filtered by splicing strength and expression significance. Abbreviations: jctn, junction; nb, neighbouring.

The complete ARH-seq splicing prediction workflow is depicted in Figure 4. The increasing depth of the sequencing machines and, thus, the growing size of RNA-seq data sets challenge all computational methods and workflows with respect to runtime efficiency. The ARH-seq workflow shows a good performance in this regard and predictions can typically be computed within few minutes for a case–control study in contrast to other methods that showed severe runtime shortfalls. The presented workflow, including mapping and counting procedures, allows processing of two sequencing lanes within 3 h. The runtime of the workflow scales linearly with the number of lanes.

### Post-processing and prioritization of splicing events

It has to be noted that all methods are influenced by factors that are not due to differential splicing, such as differential gene expression and low-level gene expression among others. It is thus recommended to employ reasonable *a posteriori* filtering of the computational predictions. Candidates displaying low expression levels could be filtered by statistically quantifying read count differences with appropriate background distributions and minimal read coverage. Such filter criteria are, however, experiment specific and should be applied in a user-defined way. In order to define reasonable splicing events we recommend filtering results according to two major criteria: expression strength and splicing indication. An exon/junction/gene is called expressed if the corresponding RPKM value is ≥0.75. In liver for example 8561 out of 49 733 genes applied to this criterion (17.2%) and 116 561 out of 446 990 exons (26.1%). In contrast, 242 740 exons had no read count at all. 72 575 junctions out of a total of 3 682 059 were expressed (2.3%) and 3 575 252 had no matching reads. To avoid artefacts based on low expression, exons should have a minimal coverage of 10 reads (123 967 exons) as well as significant differential expression. Although this cut-off is rather high and ignores the fraction of lowly expressed genes, detection of alternative splicing (in contrast to the detection of differentially expressed genes) necessitates a sufficient coverage of reads because of the complex nature of the problem so that the inclusion of lowly expressed genes would lead to a high false positive rate. Additionally, it is recommended to assess the significance of the expression differences with statistical tests that take into account specific underlying read distributions, for example a Poisson-based argument (41) resulting in 153 994 exons with *P*-value <0.01.

Splicing indication is judged with an ARH-seq *P*-value <0.2 (in the brain versus liver test case this applies for 4220 genes). Within genes with predicted splicing events exons can be ordered numerically by their splicing deviation. The exon had to be expressed in at least one of the two samples and the absolute splicing deviation was required to exceed 2.03 (27 442 exons). This value corresponded to the 95% quantile of the cumulated splicing deviation distribution. The 90 and 99% quantiles were 1.52 and 3.19, respectively.

In combination, these filters selected 899 exons in 358 genes for example for the brain versus liver case–control study. For experimental settings, for example disease versus control sample, this number will likely drop because of the higher similarity of the samples.

In order to assess the influence of sequence read length on prediction performance we used the opportunity to compare the ARH-seq results for data sets with different read lengths in Supplementary Figure S13. It can be observed that the method was robust in terms of total read number and read length. The biggest difference was due to experimental protocol differences

ARH-seq can be applied either to exon or to junction expression separately. Thus, predictions could in principle be computed separately and then joined by geometrical or statistical means and filtered for coherent events. However, in Supplementary Figure S14 we show that our approach to combine exon and junction expression before applying the splicing prediction module increased the performance drastically.

### Biological variation

While the BodyMap data used for differential splicing prediction has no biological replicates (i.e. one single lane per tissue expression experiment) ARH-seq could also be applied to scenarios with replicated experiments. This information would be incorporated into the workflow by averaging the numerical values per exon prior to the calculation of the splicing deviation for each exon (cf. Supplementary Figure S1). Since, in general, biological replicates should increase the performance of the measure, we considered the BodyMap data as a challenge for practical situations where each experimental condition is measured only once.

### Impact of mapping methods

The junction expression quantification described in this work utilizes a rather tight window for ‘synthetic’ junctions spanning two neighbouring exons so that it is likely that a large number of reads is missed. It is clear that allowing a larger window size for junction overlap increases the number of mappable reads. We exemplified this in Supplementary Figure S15. Relaxing the minimal overlap of the short reads with one of the exons to a very small extreme (5 bp) yields an increase of mappable reads over junctions to 70% and an increase in covered junctions of 28%. In addition to this ‘synthetic’ junction approach we have therefore incorporated three alternative junction mapping methods: tophat, MapSplice and SpliceMap. As illustrated in Supplementary Figures S5A and B the variation in performance is minimal when using different quantification methods. Best results were achieved with the combi-count method. However, it is also visible that the highest contribution of performance is due to exon-mapped reads and that performance based solely on junction reads is rather low. On the other hand, technology updates will increase sequencing depth and thus the coverage over junctions so that it is expectable that the impact of junction quantification for detecting differential splicing will rise.

### Missed exons

Exons have highly variable lengths ranging from a few base pairs to 18 172 bp (Figure S3A). We applied a rather stringent mapping strategy requiring the full read sequence to be aligned within an exon. However, e.g. 17.3% of the exons under analysis were shorter than 75 bp and, thus, were not included in the analysis of the ‘Illumina75’ data set. This might point to a general increasing bias of technology development that is aiming at longer sequencing reads. Here, alternative analysis schemas would be necessary that take into account partial overlap of sequence reads and exons as well as additional alignment methods allowing for example variable read length for exon quantification.

### Overlay of different isoforms

The prediction methods are designed for simple splicing events, for example one RNA that is subject to exon skipping with transcript A in sample 1 and transcript B in sample 2. A more probable scenario would be that several transcripts are processed from the same gene leading to simultaneous expression of several isoforms in the same sample eventually with different expression strengths and biological functions. If only one isoform is missing or, even more complicated, altered in one of two conditions it becomes difficult to distinguish between variances in expression and splicing variation. Since such variation also leads to gene expression changes the whole concept of ‘differential gene expression’ is questionable. Ultimately we have a correlation between isoform and function. Isoform expression change or sequence alteration could then be linked to biological effects. Due to co-expression of several isoforms with very high sequence similarities, detection and interpretation of isoform changes keeps to be an intricate challenge even with alignment based approaches and high experimental sequencing depth.

### Prediction performance

It can be seen from the ROC analyses that although the prediction performance of ARH-seq is highest among all methods it is rather small in total which accounts for the complexity of the underlying splicing prediction problem. We visualized this fact also by precision-recall curves (Supplementary Figure S16). The underlying complexity results mainly through the fact that (i) in real biological scenarios multiple isoforms are expressed simultaneously with different abundances and (ii) the knowledge on the existing isoform structures is incomplete so that we likely miss non-annotated isoforms. Furthermore, our true positive sets are still rather small and the low specificity of the methods might be explained by the fact that many true splicing effects can be found by the methods but do not account for specificity since they are not annotated within our known set of true positives. Such bias might be measured with simulation methods. On the other hand simulation data neglects all experimental bias that is not brought to the simulation model and focuses on specific parameters which can be quite different from real experiments. In a previous study we have shown with a controlled experimental set up using publicly available spike-in data at different concentrations that the ARH method performs highly accurate (18) (Supplementary Figure S17) (42).

## ACCESSION NUMBERS

GEO IDs: GSE5791, GSE12946, GSE13652 and GSE30611.

## SUPPLEMENTARY DATA

Supplementary Data are available at NAR Online.

SUPPLEMENTARY DATA
