# Supplementary material for: ARH-seq: identification of differential splicing in RNA-seq data
Source: Nucleic Acids Res. 2014 Jun 11;42(14):e110. doi: 10.1093/nar/gku495 (PMC4132698; doi:10.1093/nar/gku495)
Supplement: SUPPLEMENTARY DATA [file supp_42_14_e110__index.html]

ARH-seq: identification of differential splicing in RNA-seq data — SUPPLEMENTARY DATA 

# ARH-seq: identification of differential splicing in RNA-seq data

## SUPPLEMENTARY DATA

**Files in this Data Supplement:**

- Supplementary Data
- Supplementary Data
