# Supplementary material for: ARH-seq: identification of differential splicing in RNA-seq data
Source: Nucleic Acids Res. 2014 Jun 11;42(14):e110. doi: 10.1093/nar/gku495 (PMC4132698; doi:10.1093/nar/gku495)
Supplement: SUPPLEMENTARY DATA [file supp_gku495_nar-00692-met-g-2014-File006.pdf]

ARH-seq: Differential Splicing Prediction Workflow for RNA-seq Data

SUPPLEMENTARY MATERIAL

Axel Rasche, Matthias Lienhard, Marie-Laure Yaspo, Hans Lehrach, Ralf Herwig

Correspondence should be addressed to Axel Rasche

Institutional address

Max-Planck-Institute for Molecular Genetics
Department of Vertebrate Genomics
Bioinformatics Group
Ihnestrasse 63-73
14195 Berlin
GERMANY

Phone: +49-30-8413-1741
Fax: +49-30-8413-1740
E-mail: rasche@molgen.mpg.de, herwig@molgen.mpg.de, lienhard@molgen.mpg.de, yaspo@molgen.mpg.de, lehrach@molgen.mpg.de

A

blood2brain	162	heart2spleen	29
blood2colon	31	heart2testis	75
blood2heart	31	heart2thyroid	23
blood2kidney	53	kidney2liver	59
blood2liver	54	kidney2lung	51
blood2lung	40	kidney2muscle	69
blood2muscle	54	kidney2prostate	46
blood2prostate	29	kidney2skeletalMuscle	38
blood2skeletalMuscle	33	kidney2spleen	27
blood2spleen	38	kidney2testis	91
blood2testis	80	kidney2thyroid	45
blood2thyroid	28	liver2lung	54
brain2colon	143	liver2muscle	76
brain2heart	149	liver2prostate	51
brain2kidney	147	liver2skeletalMuscle	55
brain2liver	156	liver2spleen	40
brain2lung	162	liver2testis	98
brain2muscle	182	liver2thyroid	50
brain2prostate	159	lung2muscle	56
brain2skeletalMuscle	147	lung2prostate	33
brain2spleen	146	lung2skeletalMuscle	37
brain2testis	204	lung2spleen	32
brain2thyroid	158	lung2testis	78
colon2heart	24	lung2thyroid	32
colon2kidney	30	muscle2prostate	47
colon2liver	53	muscle2skeletalMuscle	35
colon2lung	35	muscle2spleen	56
colon2muscle	49	muscle2testis	98
colon2prostate	24	muscle2thyroid	46
colon2skeletalMuscle	28	prostate2skeletalMuscle	26
colon2spleen	15	prostate2spleen	31
colon2testis	75	prostate2testis	65
colon2thyroid	23	prostate2thyroid	21
heart2kidney	38	skeletalMuscle2spleen	35
heart2liver	47	skeletalMuscle2testis	77
heart2lung	33	skeletalMuscle2thyroid	25
heart2muscle	47	spleen2testis	78
heart2prostate	24	spleen2thyroid	30
heart2skeletalMuscle	20	testis2thyroid	72

B

blood	14
brain	111
colon	3
heart	4
kidney	10
liver	11
lung	14
muscle	27
prostate	7
skeletalMuscle	0
spleen	0
testis	54
thyroid	10

Supplementary Table S1: True Positive Sets. A: Number of true positive events selected from AEdb for the corresponding case-control study. **B:** Number of true positive events selected from AEdb listed for different tissue specific test cases.

	Illu-75 pw	Illu-75 ts	Illu-75 b2l	Illu-50f pw	Illu-50f ts	Illu-50f b2l	Illu-32 pw	Illu-32 ts	Illu-32 b2l	Affy EA pw	Affy EA ts
**ARH_combi_rpkm**	0,88	0,87	0,89	0,86	0,84	0,80	0,88	0,89	0,89	0,87	0,88
**SplicingIndex_cnt**	0,62	0,50	0,64	0,65	0,55	0,64	0,61	0,63	0,62	0,70	0,67
**PAC_combi**	0,77	0,80	0,76	0,64	0,61	0,59	0,75	0,76	0,70	0,63	0,64
**Correlation_combi**	0,80	0,76	0,64	0,80	0,72	0,78	0,82	0,82	0,68	0,77	0,82
**cuffdiff**	0,52		0,38	0,48		0,56	0,67		0,68		
**DASI_d_cnt**	0,76	0,85	0,63								
**DEXSeq**	0,70	0,67	0,57			0,68		0,62	0,61		
**MISO**	0,57		0,71	0,28		0,61					
**MATS_J**	0,53	0,53	0,66	0,51	0,54	0,53					

Supplementary Table S2: AUC for the corresponding curves in Supplementary Figure 9.
Abbrv.: pw, pairwise; ts, tissue specific; b2l, brain vs. liver; Illu, Illumina.

A

Illumina 75	ARH_combi_rpkm	SplicingIndex_cnt	PAC_combi	Correlation_combi	cuffdiff	DASI_d_cnt	DEXSeq	MISO	MATS_J
ARH_combi_rpkm	250	0.27	0.25	0.0081	0.04	0.02	0.12	0.055	0.11
SplicingIndex_cnt	105	250	0.19	0.016	0.042	0.022	0.12	0.048	0.059
PAC_combi	101	81	250	0.002	0.048	0.031	0.073	0.046	0.068
Correlation_combi	4	8	1	250	0.031	0.002	0.004	0.01	0.02
cuffdiff	19	20	23	15	250	0.075	0.062	0.075	0.13
DASI_d_cnt	10	11	15	1	35	250	0.075	0.046	0.099
DEXSeq	55	52	34	2	29	35	250	0.1	0.094
MISO	26	23	22	5	35	22	46	250	0.094
MATS_J	48	28	32	10	59	45	43	43	250

Illumina 32	ARH_combi_rpkm	SplicingIndex_cnt	PAC_combi	Correlation_combi	cuffdiff
ARH_combi_rpkm	250	0.2	0.16	0.06	0.06
SplicingIndex_cnt	82	250	0.22	0.06	0.04
PAC_combi	68	90	250	0.03	0.04
Correlation_combi	30	30	12	250	0.01
cuffdiff	26	19	21	6	250

Illumina 50f	ARH_combi_rpkm	SplicingIndex_cnt	PAC_combi	Correlation_combi	cuffdiff	MISO	MATS_J
ARH_combi_rpkm	250	0.1	0.04	0.014	0.031	0.099	0.055
SplicingIndex_cnt	46	250	0.025	0.004	0.029	0.062	0.035
PAC_combi	19	12	250	0	0.053	0.1	0.059
Correlation_combi	7	2	0	250	0.014	0.05	0.022
cuffdiff	15	14	25	7	250	0.54	0.11
MISO	45	29	47	24	176	250	0.47
MATS_J	26	17	28	11	49	159	250

Affy exon array	ARH_combi_rpkm	SplicingIndex_cnt	PAC_combi	Correlation_combi
ARH_combi_rpkm	250	0.54	0.31	0
SplicingIndex_cnt	175	250	0.3	0
PAC_combi	119	115	250	0
Correlation_combi	2	1	1	250

B

Illumina 75	ARH_combi_rpkm	SplicingIndex_cnt	PAC_combi	Correlation_combi	DASI_d_cnt	DEXSeq	MATS_J
ARH_combi_rpkm	250	0.17	0.13	0.008	0.042	0.073	0.027
SplicingIndex_cnt	73	250	0.037	0.006	0.006	0.029	0.016
PAC_combi	59	18	250	0.002	0.075	0.078	0.048
Correlation_combi	4	3	1	250	0.004	0.014	0.004
DASI_d_cnt	20	3	35	2	250	0.087	0.042
DEXSeq	34	14	36	7	40	250	0.029
MATS_J	13	8	23	2	20	14	250

Illumina 32	ARH_combi_rpkm	SplicingIndex_cnt	PAC_combi	Correlation_combi	DEXSeq
ARH_combi_rpkm	250	0.21	0.13	0.002	0.042
SplicingIndex_cnt	87	250	0.08	0.002	0.042
PAC_combi	57	37	250	0	0.035
Correlation_combi	1	1	0	250	0
DEXSeq	20	20	17	0	250

Illumina 50f	ARH_combi_rpkm	SplicingIndex_cnt	PAC_combi	Correlation_combi	MATS_J
ARH_combi_rpkm	250	0.2	0.073	0	0.02
SplicingIndex_cnt	82	250	0.025	0.004	0.014
PAC_combi	34	12	250	0	0.016
Correlation_combi	0	2	0	250	0.004
MATS_J	10	7	8	2	250

Affy exon array	ARH_combi_rpkm	SplicingIndex_cnt	PAC_combi	Correlation_combi
ARH_combi_rpkm	250	0.61	0.34	0.01
SplicingIndex_cnt	189	250	0.38	0.01
PAC_combi	128	137	250	0.01
Correlation_combi	4	3	3	250

Supplementary Table S3: A: Overlap of top 250 predictions for the heart vs. liver comparison. In the lower left triangular matrix are absolute numbers and in the upper right triangular matrix are relative overlaps following the formula $|A \cap B|/|A \cup B|$. **B:** Overlap of top 250 predictions for the liver vs. non-liver test case.

A

ARH_combi_RPKM	Illumina_75	Illumina_50f	Illumina_32	Affymetrix_EA
**Illumina_75**	250	0.02	0.28	0.26
**Illumina_50f**	10	250	0.02	0.018
**Illumina_32**	108	10	250	0.18
**Affymetrix_ExonArray**	103	9	77	250

SplicingIndex_cnt	Illumina_75	Illumina_50f	Illumina_32	Affymetrix_EA
**Illumina_75**	250	0.014	0.41	0.29
**Illumina_50f**	7	250	0.016	0.016
**Illumina_32**	146	8	250	0.26
**Affymetrix_ExonArra**	112	8	104	250

PAC_combi	Illumina_75	Illumina_50f	Illumina_32	Affymetrix_EA
**Illumina_75**	250	0.033	0.51	0.37
**Illumina_50f**	16	250	0.04	0.022
**Illumina_32**	169	19	250	0.3
**Affymetrix_ExonArra**	136	11	114	250

Correlation_combi	Illumina_75	Illumina_50f	Illumina_32	Affymetrix_EA
**Illumina_75**	250	0.062	0.037	0.025
**Illumina_50f**	29	250	0.05	0.02
**Illumina_32**	18	24	250	0.018
**Affymetrix_ExonArra**	12	10	9	250

cuffdiff	Illumina_75	Illumina_50f	Illumina_32
**Illumina_75**	250	0.12	0.11
**Illumina_50f**	55	250	0.1
**Illumina_32**	48	46	250

MISO	Illumina_75	Illumina_50f
**Illumina_75**	250	0.082
**Illumina_50f**	38	250

MATS_J	Illumina_75	Illumina_50f
**Illumina_75**	193	0.068
**Illumina_50f**	26	225

B

ARH_combi_RPKM	Illumina_75	Illumina_50f	Illumina_32	Affymetrix_EA
**Illumina_75**	250	0.2	0.26	0.12
**Illumina_50f**	83	250	0.19	0.055
**Illumina_32**	103	79	250	0.11
**Affymetrix_ExonArra**	52	26	49	250

SplicingIndex_cnt	Illumina_75	Illumina_50f	Illumina_32	Affymetrix_EA
**Illumina_75**	250	0.15	0.19	0.027
**Illumina_50f**	66	250	0.2	0.092
**Illumina_32**	80	83	250	0.059
**Affymetrix_ExonArra**	13	42	28	250

PAC_combi	Illumina_75	Illumina_50f	Illumina_32	Affymetrix_EA
**Illumina_75**	250	0.096	0.52	0.41
**Illumina_50f**	44	250	0.064	0.066
**Illumina_32**	171	30	250	0.38
**Affymetrix_ExonArra**	145	31	138	250

Correlation_combi	Illumina_75	Illumina_50f	Illumina_32	Affymetrix_EA
**Illumina_75**	250	0.062	0.062	0.014
**Illumina_50f**	29	250	0.042	0.035
**Illumina_32**	29	20	250	0.014
**Affymetrix_ExonArra**	7	17	7	250

DEXSeq	Illumina_75	Illumina_50f
**Illumina_75**	250	0.031
**Illumina_50f**	15	250

MATS_J	Illumina_75	Illumina_50f
**Illumina_75**	215	0.077
**Illumina_50f**	31	220

Supplementary Table S4: A: Overlap of top 250 predictions for the heart vs. liver test case. In the lower left triangular matrix are absolute numbers and in the upper right triangular matrix are relative overlaps following the formula $|A \cap B|/|A \cup B|$. **B:** Overlap of top 250 predictions for the liver vs. non-liver test case.

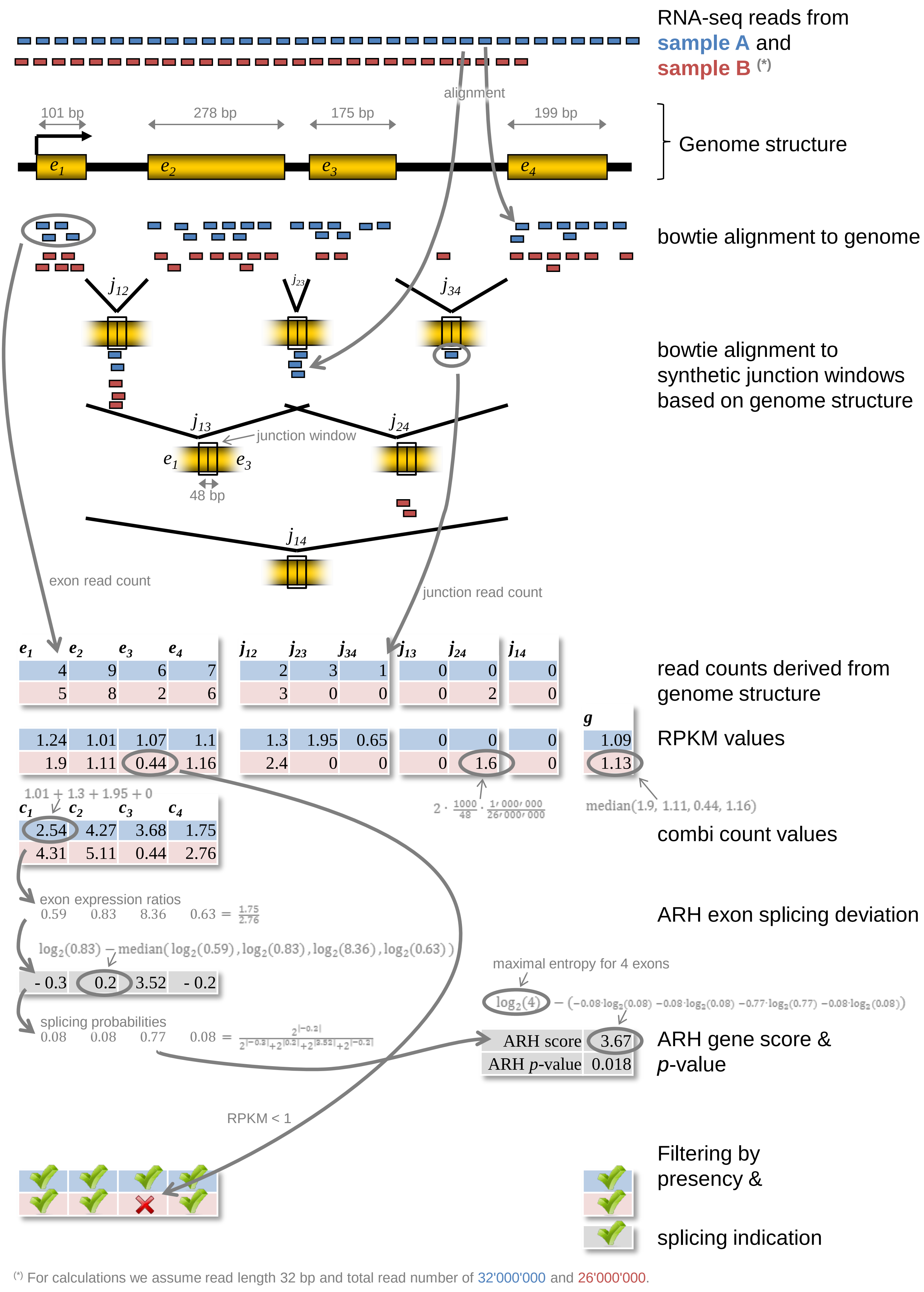

	e_1	e_2	e_3	e_4	j_{12}	j_{23}	j_{34}	j_{13}	j_{24}	j_{14}	g
Read counts A	4	9	6	7	2	3	1	0	0	0	
Read counts B	5	8	2	6	3	0	0	0	2	0	
RPKM A	1.24	1.01	1.07	1.1	1.3	1.95	0.65	0	0	0	1.09
RPKM B	1.9	1.11	0.44	1.16	2.4	0	0	0	1.6	0	1.13

	c_1	c_2	c_3	c_4
A	2.54	4.27	3.68	1.75
B	4.31	5.11	0.44	2.76
ARH exon splicing deviation	- 0.3	0.2	3.52	- 0.2

Supplementary Figure S1A: Work flow illustrating the different steps of the ARH-seq computational framework.

In the following we repeat the formal description of the method ARH as presented in (18). For a gene g with m exons, two biological conditions c and t with corresponding exon combi-counts (exon and associated junctions) $\phi_{g,e,t}$ and $\phi_{g,e,c}$, $e = 1, ..., m$, we compute the following quantities:

1. The exon splicing deviation, $\zeta_{g,e}$, measures the deviation of the fold change in each individual exon from the median gene fold change. Here, we compute log-ratios of exon fold changes to account for symmetric measurement of up- or downsplicing. From these log-ratios the median is subtracted to correct for global gene expression changes. A pseudocount of 1 on every count value avoids division by zero:

$$\zeta_{g,e} = \log_2\left(\frac{\phi_{g,e,t}+1}{\phi_{g,e,c}+1}\right) - \underset{e=1,...,m}{\text{median}}\left(\log_2\left(\frac{\phi_{g,e,t}+1}{\phi_{g,e,c}+1}\right)\right).$$

2. The exon splicing probability is computed as the absolute value of the splicing deviation $\zeta_{g,e}$ by

$$p_{g,e} = \frac{2^{|\zeta_{g,e}|}}{\sum_{e=1,...,m} 2^{|\zeta_{g,e}|}}.$$

   Note that for each gene $\sum_e p_{g,e} = 1$.

3. To measure whether the exon splicing probabilities are equally distributed or whether a single or a few exons dominate the probability distribution, we compute the entropy for each gene:

$$H_g\left(p_{g,1}, ..., p_{g,m}\right) = -\sum_{e=1}^{m} p_{g,e} \cdot \log_2\left(p_{g,e}\right).$$

4. The entropy H_g is dependent on the number of exons and can not be directly used for the comparison of different genes. Thus, in order to make the measure independent of the number of exons for a given gene, we subtract entropy from its theoretical maximum:

$$\max(H_g) - H_g = \log_2(m) - H_g\left(p_{g,1}, ..., p_{g,m}\right).$$

5. Another necessary modification accounts for the strength of deviation within the gene. This is robustly estimated with the interquartile range of exon expression ratios, the 25%, $Q_{.25,g,e=1,...,m}\left(\frac{\phi_{g,e,t}}{\phi_{g,e,c}}\right)$, and 75%, $Q_{.75,g,e=1,...,m}\left(\frac{\phi_{g,e,t}}{\phi_{g,e,c}}\right)$, quantiles. An index for the amplitude is the interquartile ratio $\frac{Q_{.75,g}}{Q_{.25,g}}$. This ratio is close to 1 for low splicing probability and increases with deviations of a number of exons in the gene. The interquartile ratio is multiplied with the entropy index and constitutes the ARH splicing prediction:

$$\text{ARH}_g = \frac{Q_{.75,g}}{Q_{.25,g}} \cdot \left(\max(H_g) - H_g\right).$$

   Thus, ARH is suitable to compare the predictions across different genes. Large ARH values (above 0.03) indicate splicing.

Supplementary Figure S1B: ARH core formula comparing two exon expression distributions.

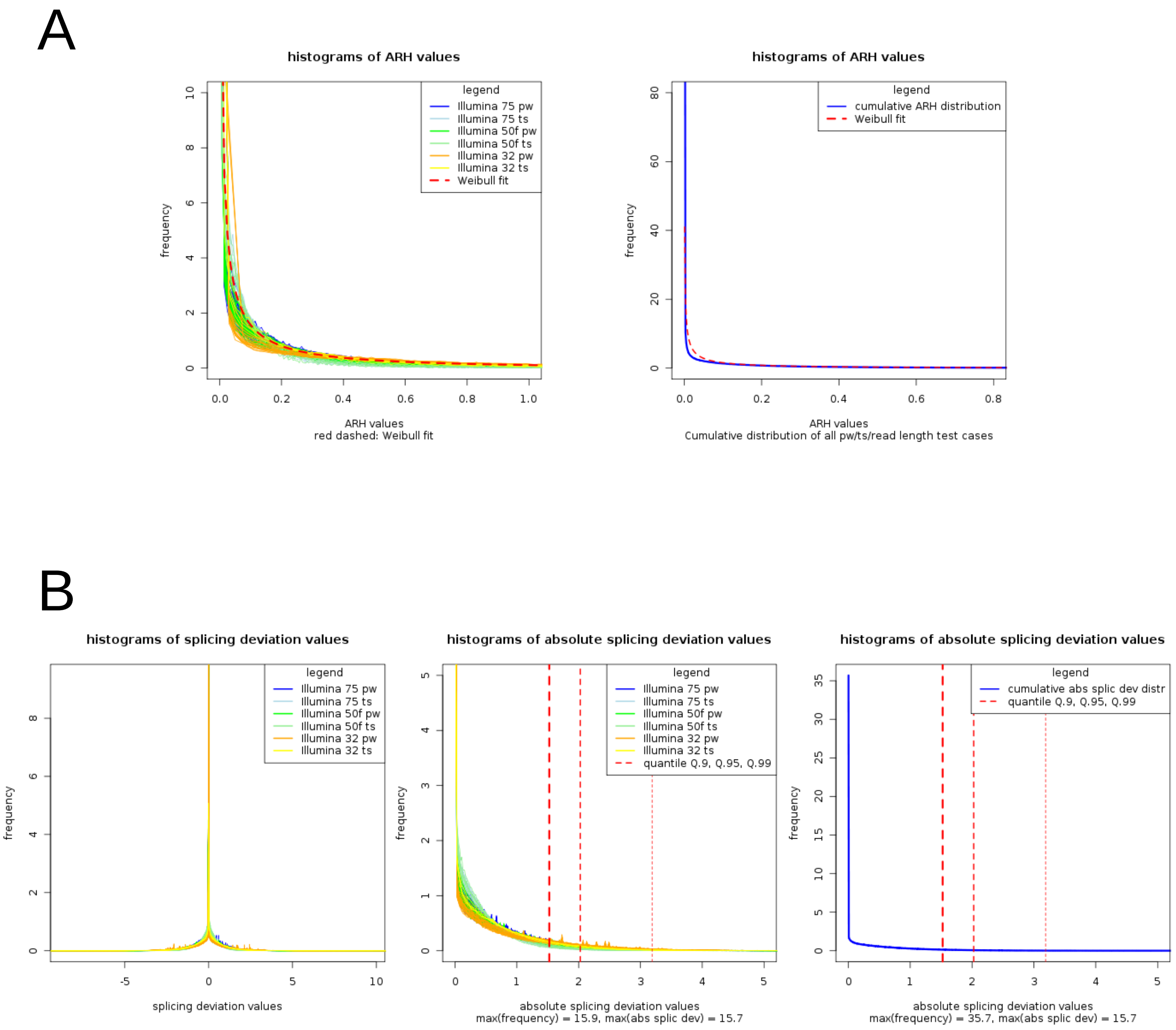

Supplementary Figure S2: A: Background distribution estimation of ARH-seq. The left plot shows ARH-seq distributions for all test cases (pairwise and tissue specific) each displayed with a different colour. The distributions show similar behaviour allowing for the estimation of a general background distribution used in different experiments. The red dotted line shows a fit with a Weibull distribution. The right plot shows the summarized ARH-seq distribution and the corresponding Weibull fit (red dotted line). **B:** Background distribution of the splicing deviations. On the left hand the splicing deviations are sign dependent with most deviations around zero. In the center image the absolute splicing deviation distributions for all the test cases (pairwise and tissue specific) are plotted, coloured by data sets. The distributions show similar behaviour allowing for the estimation of a general background distribution used in different experiments. On the right hand side all the predictions are collected for one general distribution. The vertical red lines indicate quantile cut offs at 0.9, 0.95 and 0.99 with absolute values.

A

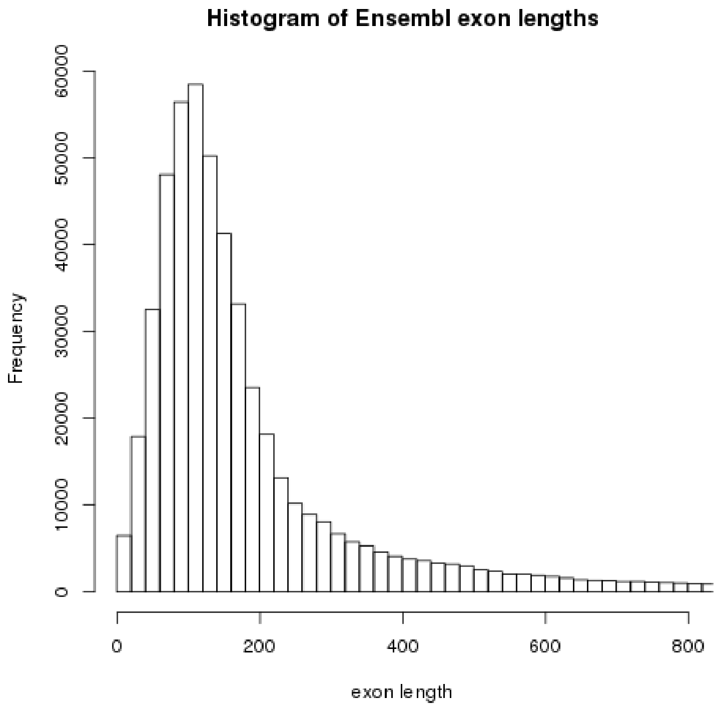

B

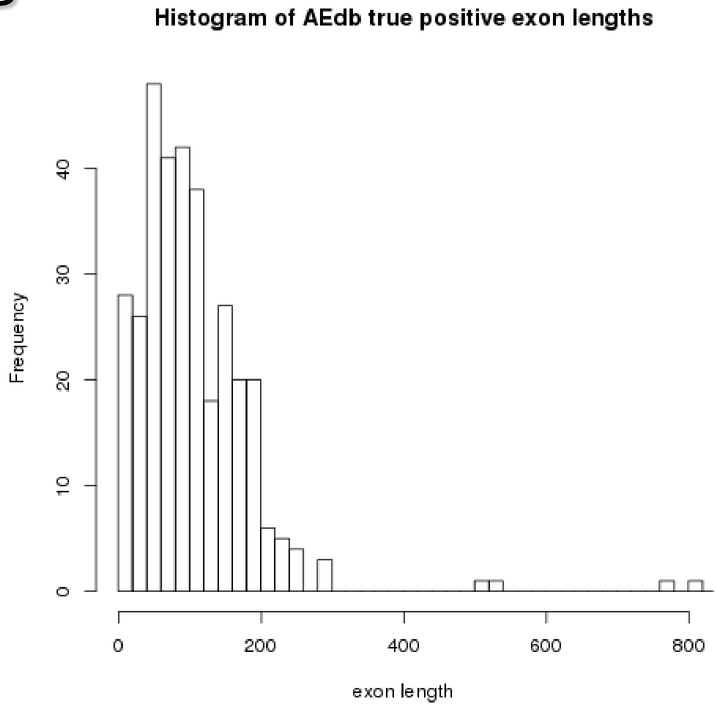

Supplementary Figure S3: Exon lengths. A: The 533’087 Ensembl exons range from 1 bp to 18’172 bp. 92’458 exons are <75 bp (17.3%) and therefore have no read count in the Illumina 75 data set. **B:** The 330 AEdb confirmed splicing events selected for tissue splicing range from 2 bp to 800 bp. 134 exons are <75 bp and therefore have no read count in the Illumina 75 data set.

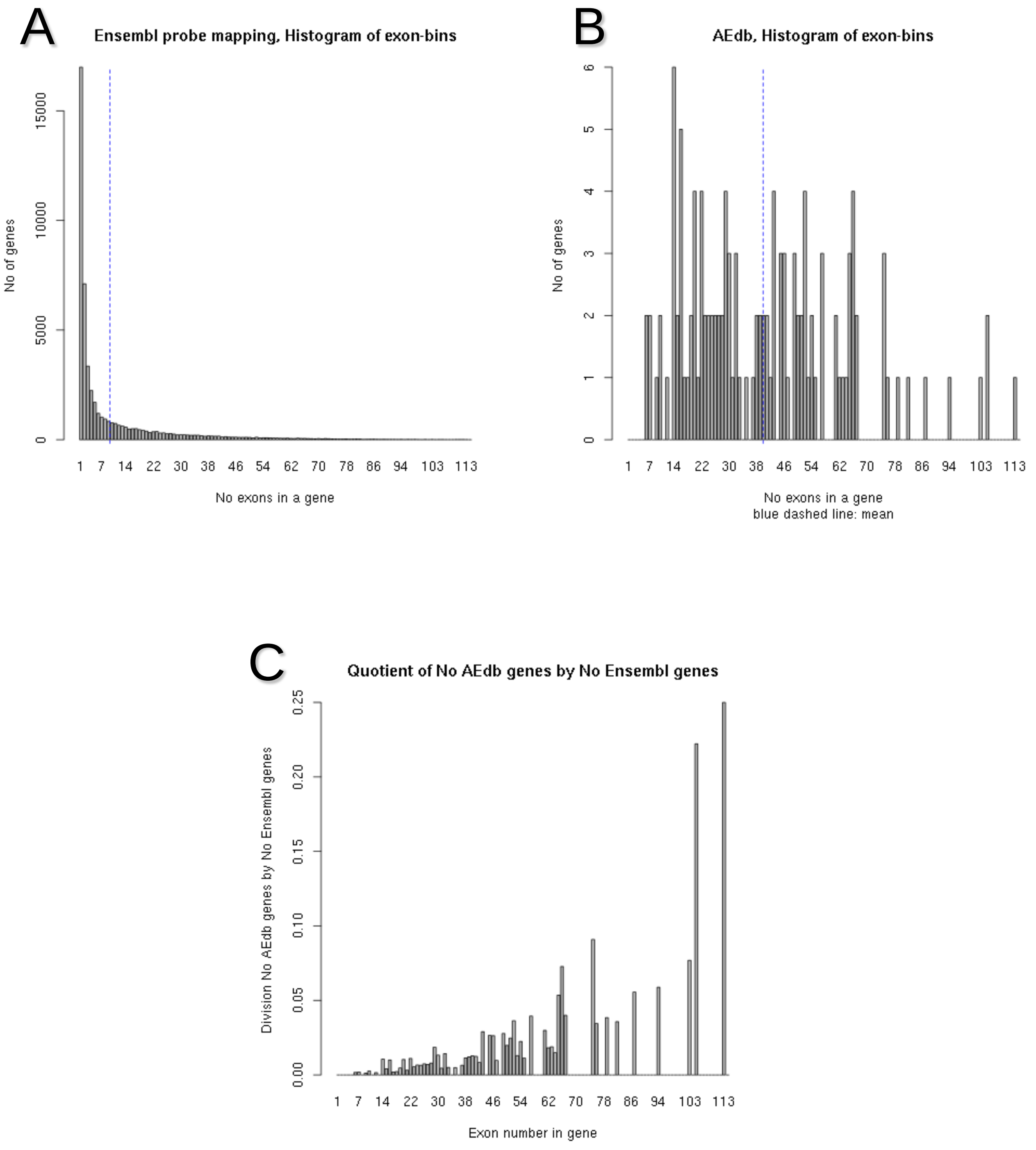

Supplementary Figure S4: A: Histogram of exon number. For all Ensembl exons used in the analysis the histogram shows that genes tend to have few exons. The blue dashed line indicates the average of 11 exons per gene. The median is 3 exons per gene. **B**: Histogram for AEdb confirmed events. The average is 47 exons per gene with a median of 40. **C**: For every exon number bin the number of genes in the AEdb histogram is divided by the number of Ensembl genes. This illustrates that genes in the AEdb are biased towards high exon numbers.

A

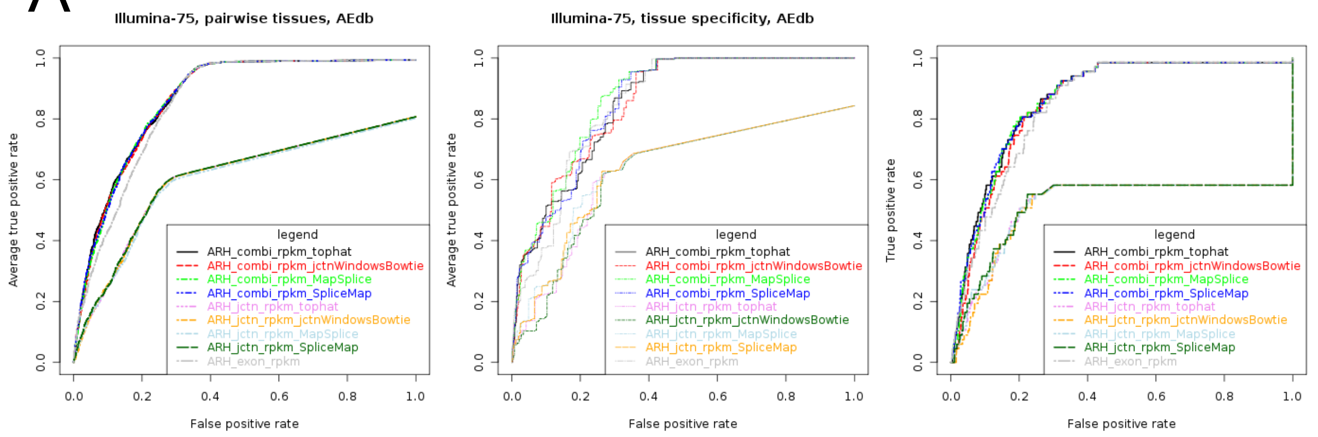

B

	pw	ts	b2l
**ARH_combi_rpkm_tophat**	0,88	0,87	0,90
**ARH_combi_rpkm_jctnWindowsBowtie**	0,88	0,86	0,89
**ARH_combi_rpkm_MapSplice**	0,89	0,88	0,90
**ARH_combi_rpkm_SpliceMap**	0,88	0,86	0,89
**ARH_jctn_rpkm_tophat**	0,57	0,57	0,51
**ARH_jctn_rpkm_jctnWindowsBowtie**	0,57	0,56	0,50
**ARH_jctn_rpkm_MapSplice**	0,57	0,59	0,51
**ARH_jctn_rpkm_SpliceMap**	0,57	0,58	0,51
**ARH_exon_rpkm**	0,88	0,88	0,89

C

	ARH_combi_rpkm_tophat	ARH_combi_rpkm_jctnWindowsBowtie	ARH_combi_rpkm_MapSplice	ARH_combi_rpkm_SpliceMap	ARH_jctn_rpkm_tophat	ARH_jctn_rpkm_jctnWindowsBowtie	ARH_jctn_rpkm_MapSplice	ARH_jctn_rpkm_SpliceMap	ARH_exon_rpkm
**ARH_combi_rpkm_tophat**	250	0,41	0,56	0,48	0,018	0,025	0,025	0,029	0,23
**ARH_combi_rpkm_jctnWindowsBowtie**	145	250	0,34	0,36	0,02	0,018	0,027	0,027	0,16
**ARH_combi_rpkm_MapSplice**	179	128	250	0,51	0,016	0,022	0,018	0,02	0,35
**ARH_combi_rpkm_SpliceMap**	162	131	168	250	0,029	0,037	0,033	0,027	0,28
**ARH_jctn_rpkm_tophat**	9	10	8	14	250	0,55	0,69	0,59	0,057
**ARH_jctn_rpkm_jctnWindowsBowtie**	12	9	11	18	177	250	0,54	0,47	0,053
**ARH_jctn_rpkm_MapSplice**	12	13	9	16	204	176	250	0,64	0,066
**ARH_jctn_rpkm_SpliceMap**	14	13	10	13	186	160	195	250	0,042
**ARH_exon_rpkm**	93	68	129	109	27	25	31	20	250

Supplementary Figure S5: Alignments and read counting. **A:** ROC curves for different junction alignment methods with respect to AEdb confirmed splicing events (Illumina 75). Junction expression is computed with tophat, MapSplice, SpliceMap and synthetic junction windows. Prediction performance is computed with ARH-seq based on junction expression, exon expression and combinations (combi counts). The left plot shows averaged pairwise tissue evaluations, the middle plot averaged tissue specific evaluations and the right plot the evaluation of the brain vs. liver scenario. Abbrv.: jctn, junction. **B:** AUC for the corresponding curves in **A**. Abbrv.: pw, pairwise; ts, tissue specific; b2l, brain vs. liver. **C:** Overlap of top 250 predictions for the brain vs. liver test case (right plot in **A**). In the lower left triangular matrix are absolute numbers and in the upper right triangular matrix are relative overlaps following the formula $|A \cap B|/|A \cup B|$.

A

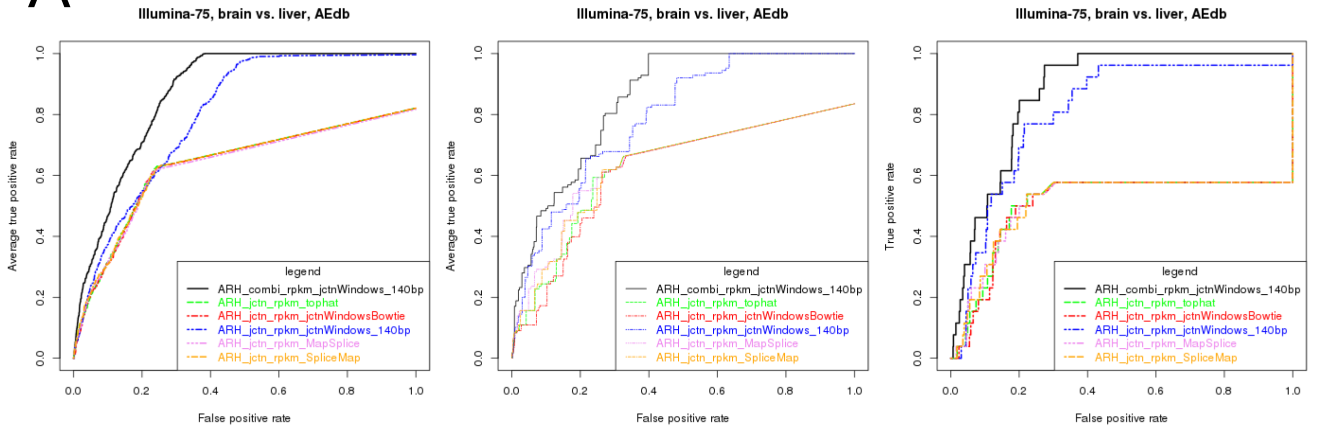

B

	pw	ts	b2l
**ARH_combi_rpkm_jctnWindows_140bp**	0,87	0,85	0,88
**ARH_jctn_rpkm_tophat**	0,57	0,57	0,51
**ARH_jctn_rpkm_jctnWindowsBowtie**	0,57	0,56	0,50
**ARH_jctn_rpkm_jctnWindows_140bp**	0,80	0,79	0,81
**ARH_jctn_rpkm_MapSplice**	0,57	0,59	0,51
**ARH_jctn_rpkm_SpliceMap**	0,57	0,58	0,51

C

	ARH_combi_rpkm_jctnWindows_140bp	ARH_jctn_rpkm_tophat	ARH_jctn_rpkm_jctnWindowsBowtie	ARH_jctn_rpkm_jctnWindows_140bp	ARH_jctn_rpkm_MapSplice	ARH_jctn_rpkm_SpliceMap	
**ARH_combi_rpkm_jctnWindows_140bp**	250	0.016	0.02	0.037	0.018	0.014	
**ARH_jctn_rpkm_tophat**	8	250	0.55	0.26	0.69	0.59	
**ARH_jctn_rpkm_jctnWindowsBowtie**	10	177	250	0.25	0.54	0.47	
**ARH_jctn_rpkm_jctnWindows_140bp**	18	102	101	250	0.26	0.23	
**ARH_jctn_rpkm_MapSplice**	9	204	176	103	250	0.64	
**ARH_jctn_rpkm_SpliceMap**	7	186	160	94	195	250	

Supplementary Figure S6: Junction prediction performance. **A:** ROC curves for different junction alignment methods with respect to AEdb confirmed splicing events (Illumina 75). Junction expression is computed with tophat, MapSplice, SpliceMap and synthetic junction windows. Prediction performance was computed with ARH-seq based on junction/combi-count expression and synthetic junctions with sizes 112 bp and 140 bp. The left plot shows averaged pairwise tissue evaluations, the middle plot averaged tissue specific evaluations and the right plot the evaluation of the brain vs. liver scenario. Abbrv.: jctn, junction. **B:** AUC for the corresponding curves in **A**. Abbrv.: pw, pairwise; ts, tissue specific; b2l, brain vs. liver. **C:** Overlap of top 250 predictions for the brain vs. liver test case (right plot in **A**). In the lower left triangular matrix are absolute numbers and in the upper right triangular matrix are relative overlaps following the formula $|A \cap B| / |A \cup B|$.

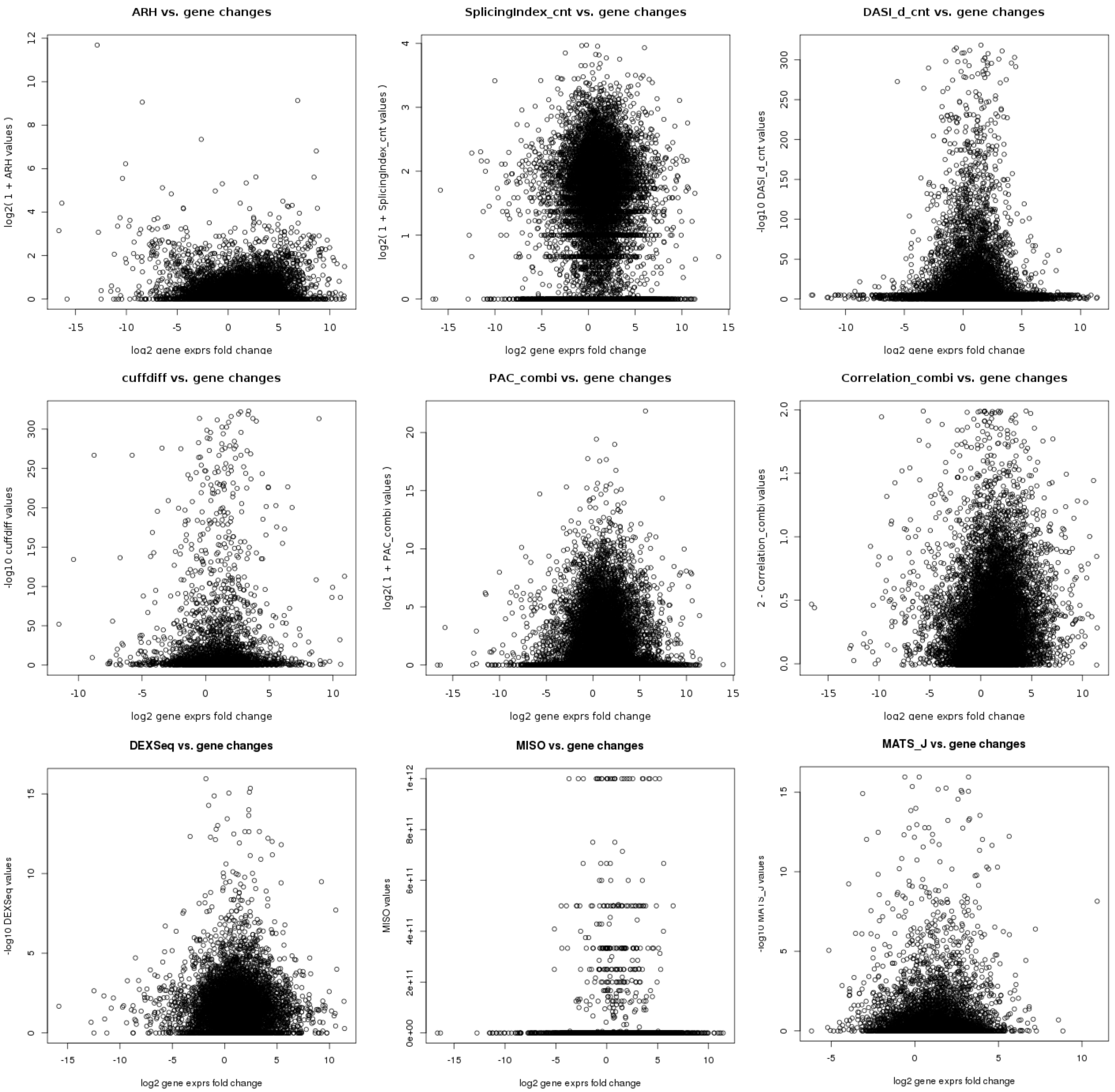

Supplementary Figure S7: Differential splicing vs. differential expression. Splicing prediction is plotted vs. gene expression fold-changes in brain vs. liver (Illumina 75). To account for increased and decreased gene expression changes, log-expression values are provided on the *x*-axes. Splicing predictions on the *y*-axes are always positive and on logarithmised scale except DASI. It provides *p*-values and is here visualised on $-\log_{10}$ scale.

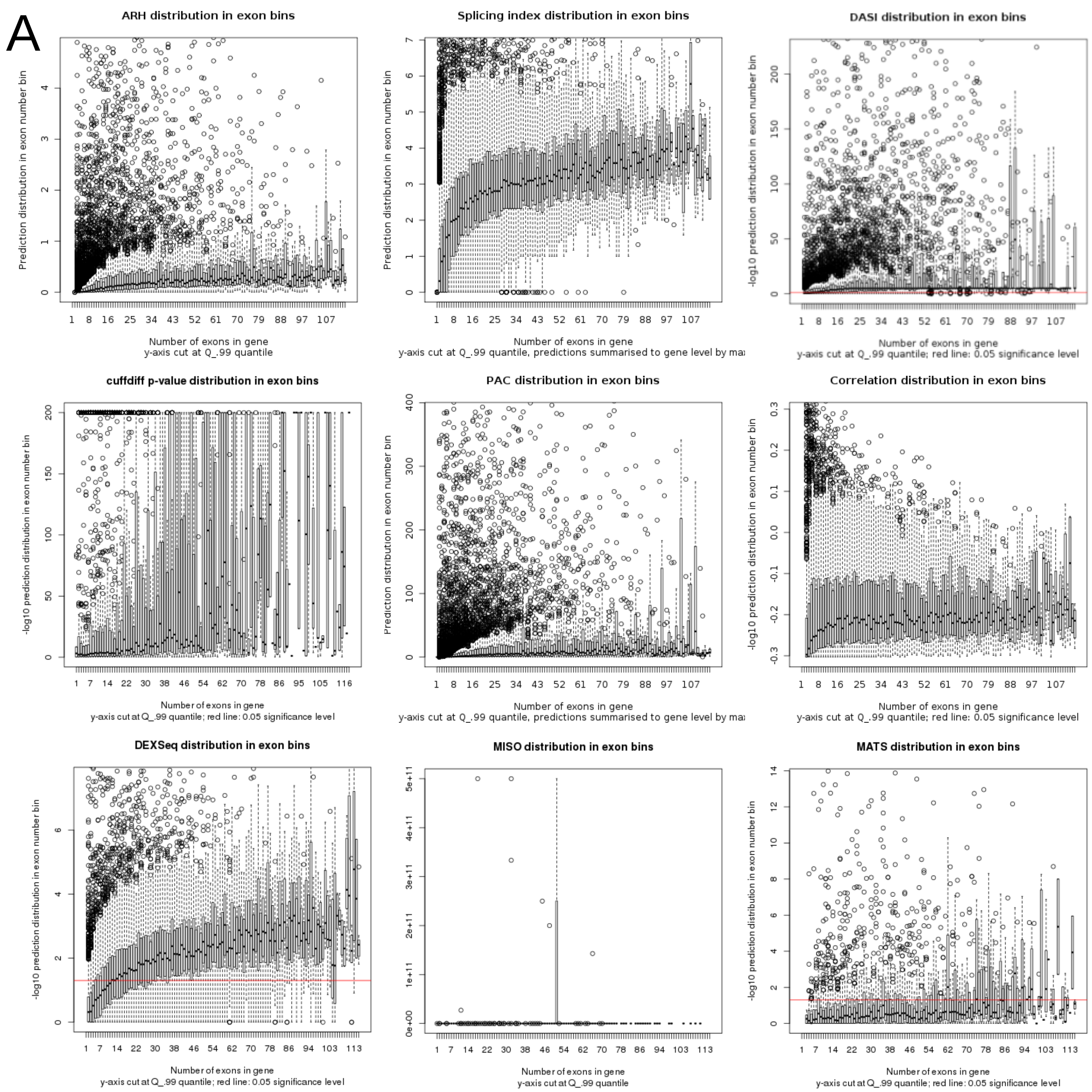

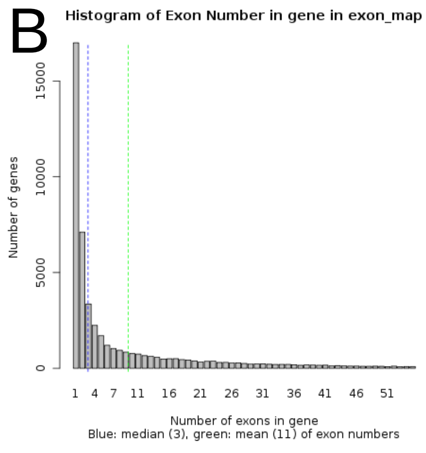

Supplementary Figure S8: Dependency of differential splicing prediction on exon number. **A:** Splicing prediction is plotted vs. exon number. Predictions are binned by the exon number. For each exon number bin a box-and-whiskers plot is displayed. A horizontal red line indicates the *p*-value 0.05 threshold line. The graphics show exon number dependency of predictions unaware of any true positive splicing events. **B**: Histogram of the exon numbers in Ensembl 58.

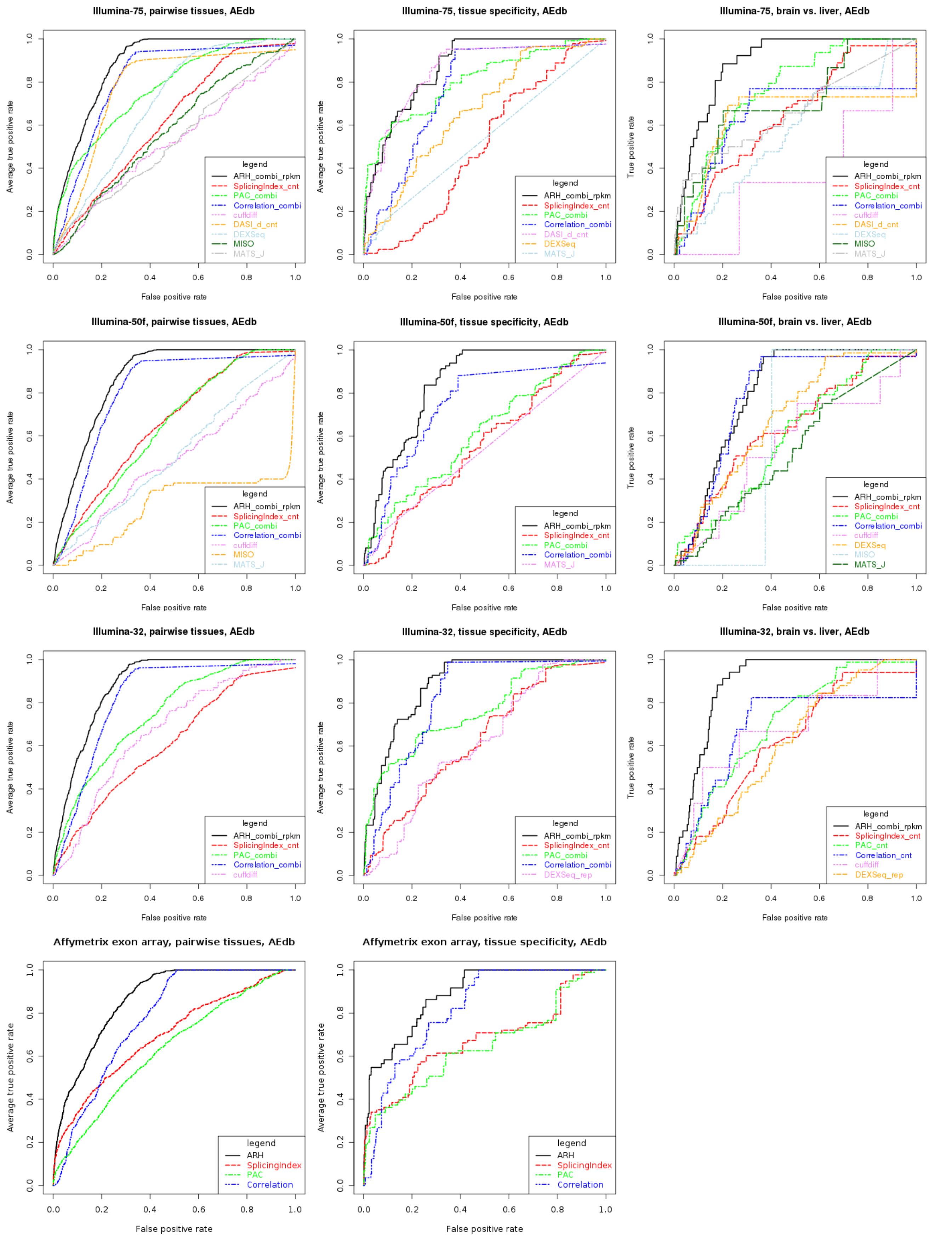

Supplementary Figure S9: Splicing prediction performance. Splicing prediction methods are compared on various tisse data sets. The left panel shows averaged pairwise tissue evaluations, the center panel averaged tissue specific evaluations and the right panel the evaluation of the brain vs. liver scenario. Columns show Illumina 75, Illumina 50f, Illumina 32 and the Affymetrix exon array data sets. Brain tissue is not available for exon arrays, the respective graphic is thus skipped. The MISO method uses for Illumina-50 the paired end feature. DEXSeq uses a work-around for the non-replicative data sets, except for Illumina-32.

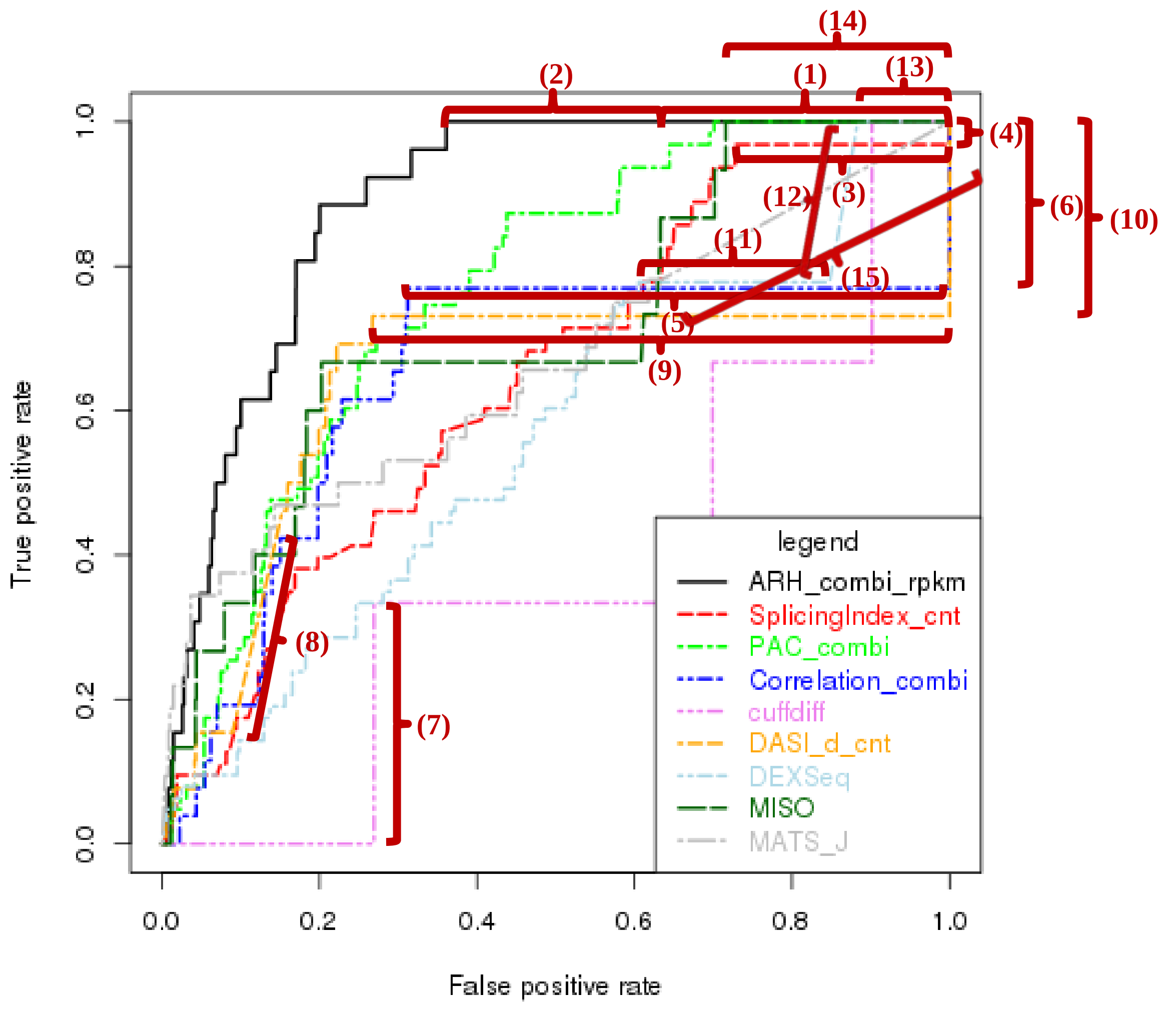

Supplementary Figure S10: Artefacts description. The method results are ordered by decreasing splicing indication. Depending on the strengths and drawbacks of the methods visible effects influence methods performance. For the Illumina 75 test case brain vs. liver tissue the effects are explained here:

(1)[ARH_combi_rpkm] No predictions available, e.g. genes have not enough exons or no finite ratios.

(2)[ARH_combi_rpkm] Interpolation over many predictions with low indication, i.e. no splicing indication.

(3)[SplicingIndex_cnt] No predictions available. When no read is found for an exon this leads to division by zero or non-finite values.

(4)[SplicingIndex_cnt] If (3), no prediction available, applies to true positive events, those are counted last. With the final predictions the curve climbs to the upper right. This corresponds to a penalty function, if predictions for true positive events are not possible.

(5)[Correlation_combi] No predictions available, e.g. genes have not enough exons with finite non-zero values to compute a correlation value.

(6)[Correlation_combi] If, no prediction is available (see (6)) for true positive events, those are counted last.

(7)[cuffdiff] For most genes and true positives no predictions are available due to low read coverage of the genes/transcripts. All these genes are skipped. Thus performance is rated with available true positives leading to bigger steps in the curve.

(8)[DASI_d_cnt] Same DASI *p*-value applies to many genes (24 TP genes in this case) due to the Fisher test.

(9)[DASI_d_cnt] No predictions available, e.g. not enough exons to compute the Fisher test.

(10)[DASI_d_cnt] If no prediction is available (see (11)) for true positive events, those are counted last.

(11)[DEXSeq] Many non-TP predictions with very low indication, i.e. *p*-value > 0.99996

(12)[DEXSeq] Interpolation over many predictions with low indication, i.e. no splicing indication.

(13)[DEXSeq] No predictions available.

(14)[MISO] Many non-TP predictions with very low indication.

(15)[MATS_J] Interpolation over many predictions with low indication, i.e. no splicing indication.

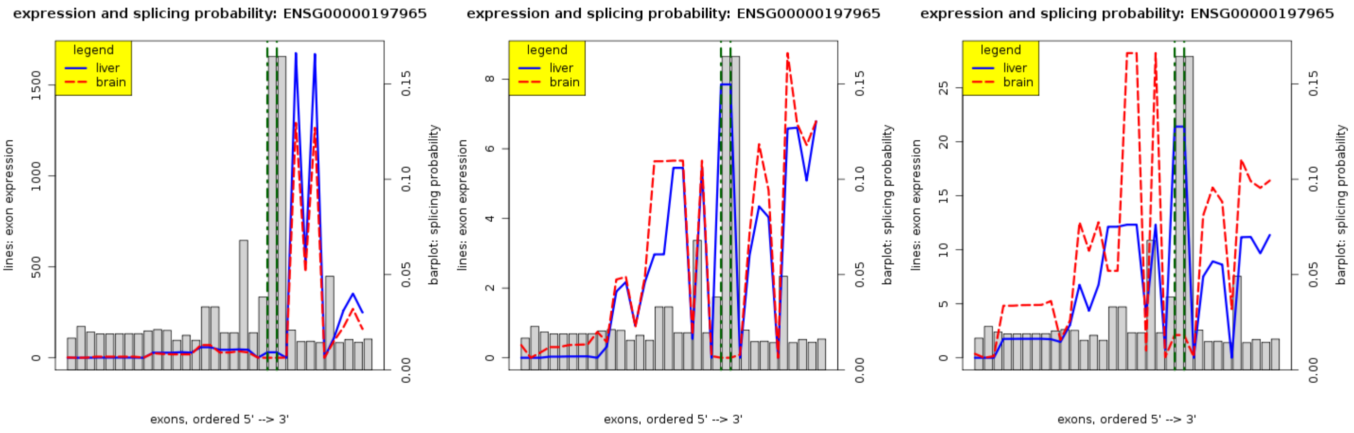

raw count RPKM values combi counts

Supplementary Figure S11: Selected example *MPZL1*. Exon expression behaviour over exons genomically ordered for the gene *MPZL1* (Zhao and Zhao 2003). 'treat' corresponds to brain (red, dashed) and 'ctrl' to liver tissue (blue). Exon expression, basis for the predictions, are refined from left to right starting with raw exon read counts with RPKM scaling to combi counts including junction expression. The grey bar plots visualize the splicing probabilities for the exons, basis for the splicing assessment with entropy. The green dot-dashed line marks the two true positive tissue splicing events known for the gene.

A

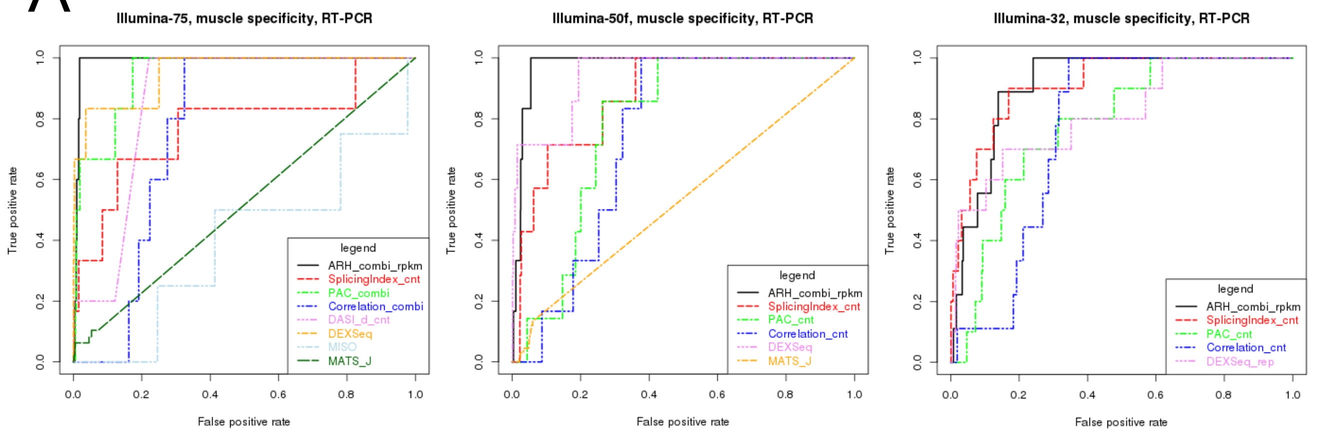

B

	Illu-75	Illu-50f	Illu-32
**ARH_combi_rpkm**	0,99	0,98	0,91
**SplicingIndex_cnt**	0,77	0,88	0,91
**PAC_cnt**	0,94	0,78	0,78
**Correlation_cnt**	0,76	0,75	0,76
**DASI_d_cnt**	0,86		
**DEXSeq**	0,95	0,94	0,81
**MISO**	0,40		
**MATS_J**	0,52	0,54	

C

	ARH_combi_rpkm	DASI_d_cnt	SplicingIndex_cnt	PAC_cnt	Correlation_cnt	DEXSeq	MISO	MATS_J
**ARH_combi_rpkm**	250	0.17	0.12	0	0.031	0.096	0.035	0.059
**DASI_d_cnt**	72	250	0.031	0.004	0.002	0.022	0.016	0.006
**SplicingIndex_cnt**	55	15	250	0	0.053	0.068	0.059	0.055
**PAC_cnt**	0	2	0	250	0.002	0.006	0.002	0.002
**Correlation_cnt**	15	1	25	1	250	0.04	0.012	0.044
**DEXSeq**	44	11	32	3	19	250	0.025	0.055
**MISO**	17	8	28	1	6	12	250	0.037
**MATS_J**	28	3	26	1	21	26	18	250

Supplementary Figure S12: A: Method evaluation with muscle-specific exon skipping events, RT-PCR. ROC curves for different muscle vs. non-muscle test cases. The validated events were predicted on Affymetrix exon array tissue data with Splicing Index and MiDAS. Validation was performed with RT-PCR. From left to right are the evaluations for Illumina 75, 50f and 32 data sets. Abbrv.: cnt, count. **B:** Method evaluation with muscle-specific exon skipping events, RT-PCR. AUC for the corresponding curves in A. Abbrv.: Illu, Illumina. **C:** Method evaluation with muscle-specific exon skipping events, RT-PCR. Overlap of top 250 predictions for muscle vs. non-muscle test case Illumina 75 data set (left hand graphic in supplementary Figure 6). In the lower left triangular matrix are absolute numbers and in the upper right triangular matrix are relative overlaps following the formula $|A \cap B| / |A \cup B|$.

A

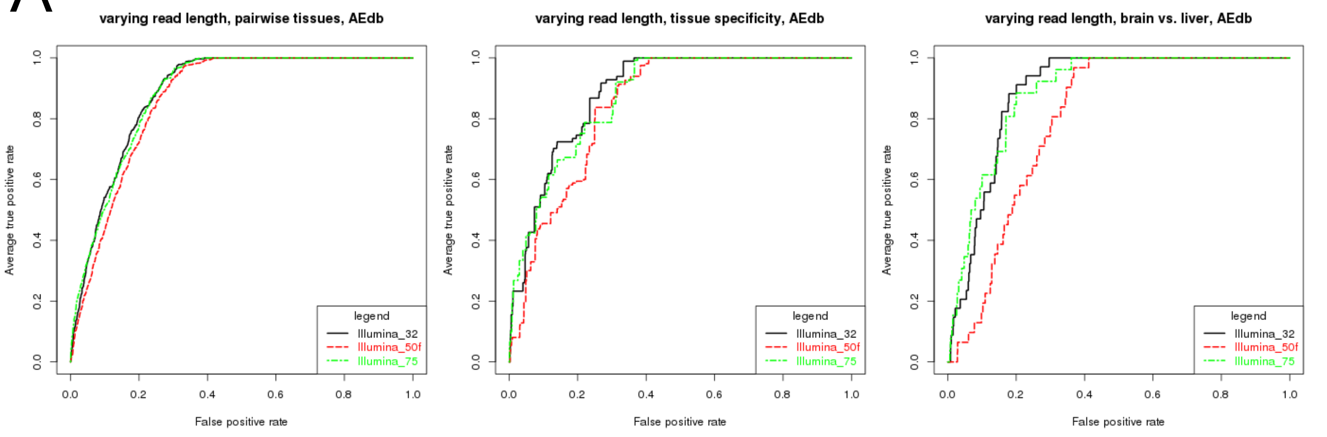

B

	pw	ts	b2l
**Illumina 32**	0.88	0.89	0.89
**Illumina 50f**	0.86	0.84	0.80
**Illumina 75**	0.88	0.87	0.89

Supplementary Figure S13: A: Read length evaluation. ROC curves for ARH-seq predictions on data sets with varying read length. The Illumina 50f data set aligns only forward reads from the paired-end protocol. On the left hand side are averaged pairwise tissue evaluations, in the center averaged tissue specific evaluations and on the right hand side the evaluation of the brain vs. liver scenario. **B:** Read length evaluation. AUC for the corresponding curves. Abbrv.: pw, pairwise; ts, tissue specific; b2l, brain vs. liver.

A

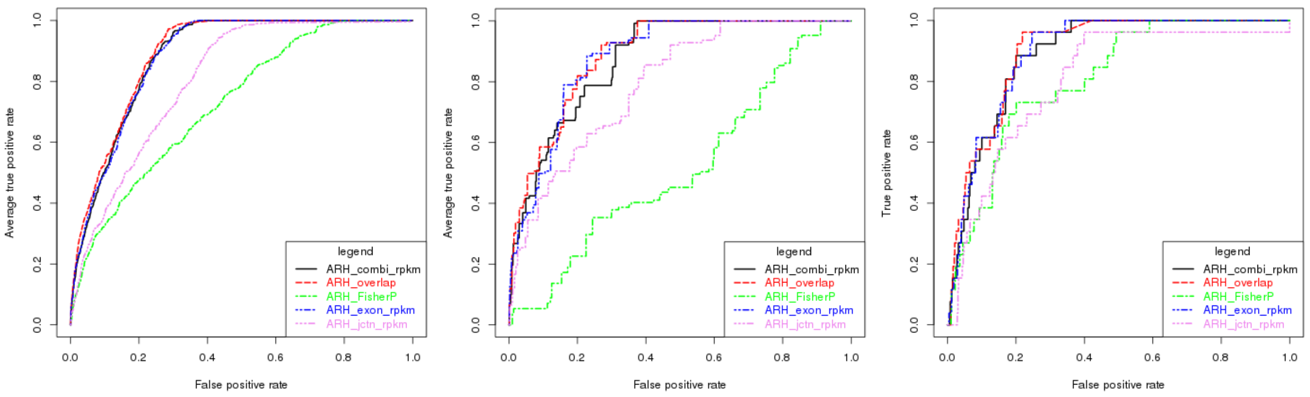

B

	pw	ts	b2l
**ARH_combi_rpkm**	0.88	0.87	0.89
**ARH_overlap**	0.89	0.89	0.90
**ARH_FisherP**	0.73	0.52	0.81
**ARH_exon_rpkm**	0.88	0.88	0.89
**ARH_jctn_rpkm**	0.81	0.79	0.80

C

	ARH_combi_rpkm	ARH_overlap	ARH_FisherP	ARH_exon_rpkm	ARH_jctn_rpkm
**ARH_combi_rpkm**	250	0.26	0.23	0.33	0.096
**ARH_overlap**	102	250	0.31	0.48	0.24
**ARH_FisherP**	95	117	250	0.32	0.24
**ARH_exon_rpkm**	124	162	120	250	0.16
**ARH_jctn_rpkm**	44	98	98	68	250

Supplementary Figure S14: A: ROC curves for different exon-junction consensus methods (Illumina 75). ARH-seq predictions are computable for exons and junctions separately. Here, we evaluate the best method to join the predictions of the two data types. Possible approaches are (1) overlap, (2) Fisher-P and (3) combining expression values in beforehand to splicing prediction. In (1) the lower prediction value of exon or junction prediction is selected. In (2) *p*-values of the two predictions are combined via the χ^2-test, also named Fisher-P approach (Weibull parameters fitted to exon based ARH-seq background distribution). Both approaches are incriminated by low junction-based prediction performance. In (3) we propose to combine exon and junction expression in beforehand in the so-called combi counts and then run ARH-seq as prediction method. Junction predictions are generally hindered by the fact, that for many genes no junction reads are aligned at all. On the left hand side are averaged pairwise tissue evaluations, in the center averaged tissue specific evaluations and on the right hand side the evaluation of the brain vs. liver scenario. Abbrv.: jctn, junction. **B:** AUC for the corresponding curves in A. Abbrv.: pw, pairwise; ts, tissue specific; b2l, brain vs. liver. **C:** Overlap of top 250 predictions for the brain vs. liver test case (right hand graphic in A). In the lower left triangular matrix are absolute numbers and in the upper right triangular matrix are relative overlaps following the formula $|A \cap B| / |A \cup B|$.

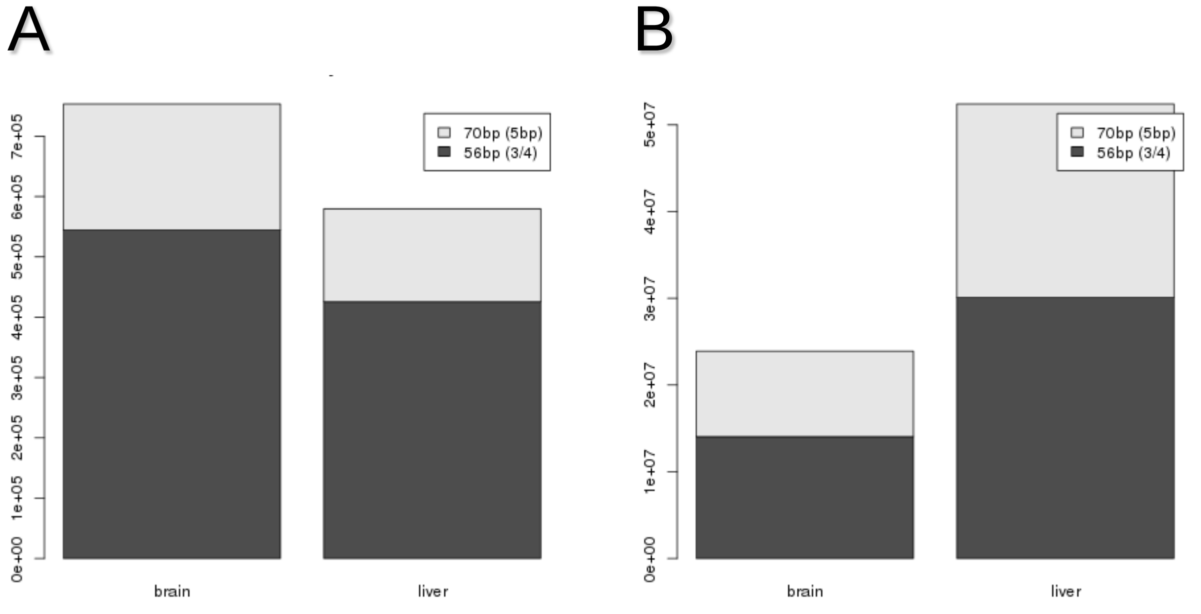

Supplementary Figure S15: Effect of synthetic junction sizes. **A:** Expecting ¼ read length overlap 544’250 junctions are covered with at least one read in brain. Relaxing this overlap to a minimum of 5 bp, 753’416 junctions are covered, a 27.7% increase in coverage. **B:** At the same time with ¼ read length overlap 14’036’931 reads are aligned to junctions in brain and 23’898’512 reads for the smaller overlap. This corresponds to a 70.3% increase in aligned reads.

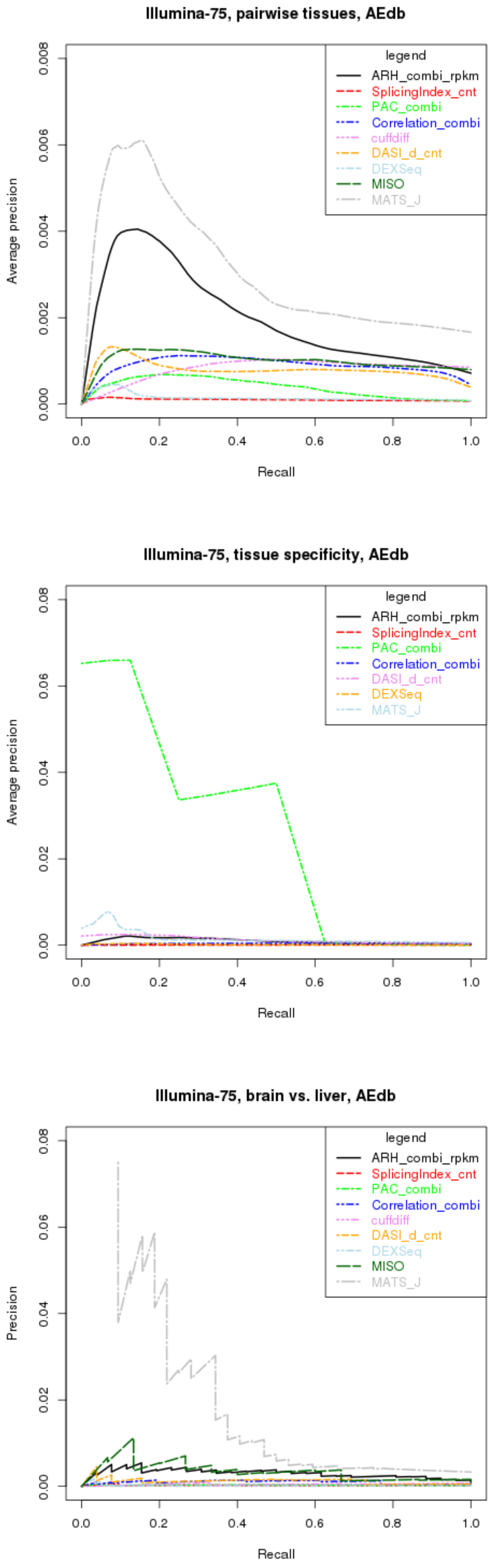

Supplementary Figure S16: Precision-Recall plots for the Illumina-75 data set.

A

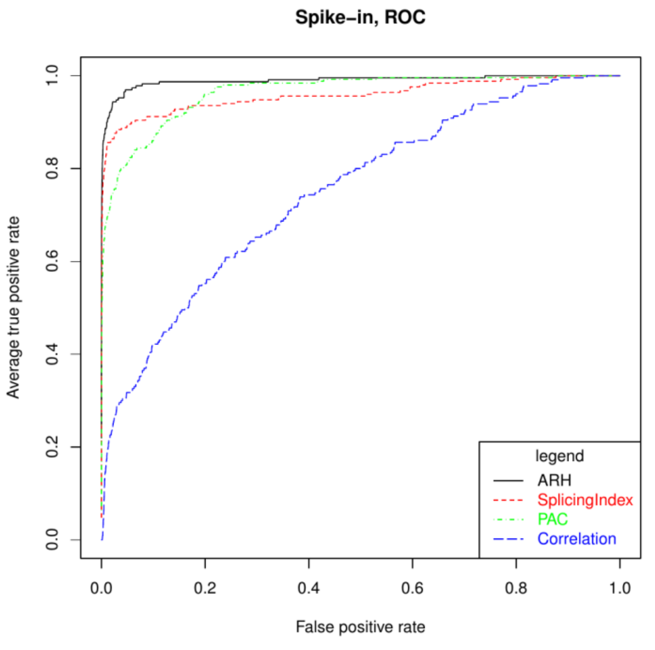

B

	Spike-in
**ARH**	0,99
**SplicingIndex**	0,96
**PAC**	0,96
**Correlation**	0,75

Supplementary Figure S17: Spike-in exon array performance. In Abdueva et al. (2007), a benchmark dataset was presented with spike-in transcripts. In HeLa cells 25 non-expressed transcripts are added at different concentrations in a Latin square design by five groups. Following the original handling of the data, we used the Affymetrix probe–probe set-transcript cluster assignment. Exons are re-assigned to different transcripts to establish generic splicing events. The environment excluding the 25 transcripts has no expression change at low variability. The samples are hybridized on the arrays in triplicates.
